# Epigenetic Dysregulation Induces Translocation of Histone H3 into Cytoplasm

**DOI:** 10.1002/advs.202100779

**Published:** 2021-08-07

**Authors:** Zhen Wang, Ji Chen, Chuan Gao, Qiong Xiao, Xi‐Wei Wang, Shan‐Bo Tang, Qing‐Lan Li, Bo Zhong, Zhi‐Yin Song, Hong‐Bing Shu, Lian‐Yun Li, Min Wu

**Affiliations:** ^1^ College of Life Sciences Wuhan University Wuhan 430072 China; ^2^ Hubei Key Laboratory of Cell Homeostasis Hubei Key Laboratory of Developmentally Originated Disease Hubei Key Laboratory of Enteropathy Wuhan University Wuhan 430072 China; ^3^ Frontier Science Center for Immunology and Metabolism Wuhan University Wuhan 430072 China; ^4^ Department of Immunology Medical Research Institute School of Medicine Wuhan University Wuhan 430071 China

**Keywords:** autophagy, cGAS, H3K9me3, heterochromatin, *HP1α*, SETDB1

## Abstract

In eukaryote cells, core components of chromatin, such as histones and DNA, are packaged in nucleus. Leakage of nuclear materials into cytosol will induce pathological effects. However, the underlying mechanisms remain elusive. Here, cytoplasmic localization of nuclear materials induced by chromatin dysregulation (CLIC) in mammalian cells is reported. H3K9me3 inhibition by small chemicals, *HP1α* knockdown, or knockout of H3K9 methylase SETDB1, induces formation of cytoplasmic puncta containing histones H3.1, H4 and cytosolic DNA, which in turn activates inflammatory genes and autophagic degradation. Autophagy deficiency rescues H3 degradation, and enhances the activation of inflammatory genes. *MRE11*, a subunit of MRN complex, enters cytoplasm after heterochromatin dysregulation. Deficiency of *MRE11* or *NBS1*, but not *RAD50*, inhibits CLIC puncta in cytosol. *MRE11* depletion represses tumor growth enhanced by *HP1α* deficiency, suggesting a connection between CLIC and tumorigenesis. This study reveals a novel pathway that heterochromatin dysregulation induces translocation of nuclear materials into cytoplasm, which is important for inflammatory diseases and cancer.

## Graphical abstract

The current study reveals a novel pathway leading to cytoplasmic localization of nuclear material induced by chromatin dysregulation (CLIC). *HP1α* knockdown, SETDB1 knockout or H3K9me3 inhibition by drugs, induces cytoplasmic puncta containing histones H3.1, H4 and DNA, which is regulated by *MRE11* and *NBS1*. CLIC activates inflammatory gene expression, *γ*H2AX formation, and autophagy, and is related with inflammation and tumorigenesis.

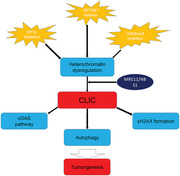

## 1 Introduction

In eukaryotic cells, DNA binds on nucleosomes to form chromatin which is packaged inside of nuclear membrane. Translocation of chromatin DNA into cytoplasm has been linked with severe consequences such as cancer and autoimmune diseases.^[^ ^1^ ^]^ But the mechanisms controlling the translocation are not clear. Innate immunity pathways sensing intracellular viral DNA, such as cGAS/TBK1 pathway, can be activated by cytosolic DNA translocated from nuclei and induce inflammation.^[^ ^2^ ^]^ Meanwhile, it is believed cytoplasmic DNA is associated with chromosomal instability and metastasis.^[^ ^3^ ^]^ Some studies have shown that senescence and telomere damage induce autophagic degradation of nuclear proteins in mammalian cells.^[^ ^4^, ^5^ ^]^ These indicate that core nuclear components can be translocated into cytosol during certain circumstance and may have pathological consequences. However, it is not clear how the process is regulated and which signals can initiate the process.

In mammalian cells, heterochromatin is critical for chromatin stability, repressing gene expression and transposon activity. Heterochromatin is usually marked with trimethylation of histone H3 lysine 9 (H3K9me3) or trimethylation of histone H3 lysine 27 (H3K27me3). Suppressor of variegation 3‐9 homolog 1/2 (SUV39H1/2) are the main methylases for H3K9me3, and SET domain bifurcated histone lysine methyltransferase 1 (SETDB1) is the enzyme for transposon regions, especially endogenous retroviruses.^[^ ^6^, ^7^ ^]^ Heterochromatin 1 (HP1) then binds to H3K9me3 and condenses chromatin. ^[^ ^8^ ^]^ H3K9me3 deficiency has been associated with abnormal gene expression and increased mutations in cancer cells.^[^ ^9^, ^10^ ^]^ However, it still requires further work to study whether H3K9me3 deficiency is related with other consequences during tumorigenesis.

Epigenetic factors have also been shown to be involved in the regulation of autophagy.^[^ ^11^ ^]^ H4K16ac is involved in the regulation of autophagic gene expression;^[^ ^11^, ^12^ ^]^ and histone H1.2 has been shown to regulate autophagy in the diabetic retinopathy.^[^ ^13^ ^]^ Small chemical inhibitors for epigenetic enzymes, such as BIX‐01294, an inhibitor of histone H3K9 methylation and 2‐PCPA, an inhibitor of H3K4 demethylase lysine demethylase 1A (KDM1A, also known as LSD1), have been shown to induce autophagy in mammalian cells,^[^ ^14^, ^15^, ^16^, ^17^ ^]^ but the underlying mechanisms are not clear.

Previously, we have discovered that several inhibitors of epigenetic enzymes induce autophagy in mammalian cells.^[^ ^15^ ^]^ In the current study, we surveyed these inhibitors and the related enzymes, and found that down regulation of H3K9 methylation induced cytosolic localization of DNA, histones H3.1 and H4, which consequently activated cGAS pathway and was degraded by autophagy. SETDB1 and *HP1α* deficiency mimicked the phenotype, suggesting a potential link with its function on transposon repression. Our study implies a novel pathway which might be critical for cancer and autoimmune diseases in mammalian cells.

## 2 Results

### 2.1 Down Regulation of H3K9 Methylation Induces Cytosolic Histone H3

**Figure 1:**
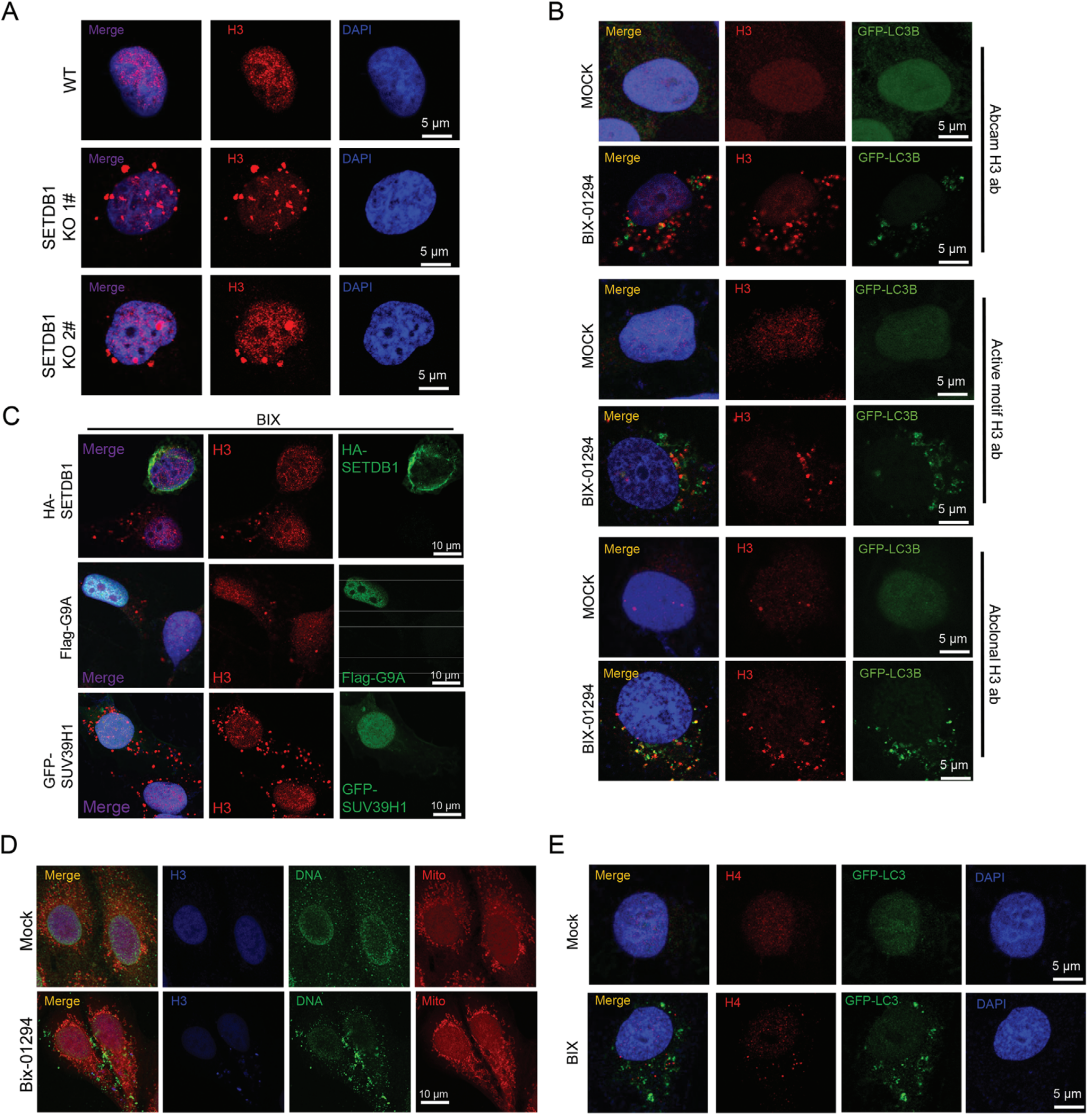
Inhibition of H3K9me3 induces cytosolic localization of histone H3. A) SETDB1 was knocked out in U2OS cells and histone H3 were analyzed by confocal microscopy. Scale bar, 5 µm. B) GFP‐LC3B‐U2OS Cells were treated with 5 µm BIX‐01294 for 8 h and H3 was studied with confocal microscopy. Three different H3 antibodies were used. Scale bar, 5 µm. C) HA‐SETDB1, Flag‐G9A, and GFP‐SUV39H1 were transfected into U2OS cells and treated with 5 µm BIX‐01294 for 8 h. The indicated proteins were imaged with confocal microscope. Scale bar, 10 µm. D) DNA was stained by anti‐DNA antibody and studied with confocal microscopy. Mitochondria were labeled with Mito‐tracker (Invitrogen, M7521). Cells were treated with 5 µm BIX‐01294 for 8 h. E) GFP‐LC3‐U2OS cell were treated with 5 µm BIX‐01294 for 12 h. Histone H4 was imaged with confocal microscopy. Scale bar, 5 µm.

In a survey of enzymes for histone modifications, we found that the defect of SETDB1, a known methylase for H3K9me3, caused H3 puncta outside of nucleus (**Figure** **1**A; Figure S1A, Supporting Information). Since SETDB1 catalyzes H3K9 methylation, we applied one widely‐used chemical inhibitor for H3K9 methylation, BIX‐01294, to U2OS cells. Since BIX‐01294 can induce autophagic cell death,^[^ ^14^, ^16^, ^18^ ^]^ we confirmed its effect in repressing H3K9 methylation and tested cell toxicity, then used 5 µm in most of the following studies, which did not induce cell death in 48 h (Figure S1B,C, Supporting Information). We found that BIX‐01294 also induced cytosolic H3 similar to SETDB1 knockout (Figure 1B). To exclude the possibility of antibody cross activity, we applied three antibodies for H3 from different merchants and got the same results (Figure 1B). To investigate whether other methylases for H3K9 methylation are also involved in the process, we knocked down euchromatic histone lysine methyltransferase 2 (EHMT2/G9a) and SUV39H1 in U2OS cells, respectively. Neither G9a nor SUV39H1 deficiency induced cytoplasmic H3 (Figure S1D,E, Supporting Information). Exogenous expression of SETDB1 inhibited the cytoplasmic H3 induced by BIX‐01294, but not G9a or SUV39H1 (Figure 1C; Figure S1F, Supporting Information). These suggest the phenotype of cytosolic H3 puncta is closely related with SETDB1.

### 2.2 DNA, H4, and S10‐Phosphorylated H3 were Translocated into Cytoplasm

To study whether other nuclear materials appeared in cytosol after BIX‐01294 treatment, we performed immunostaining for DNA and modified histones. Chemical dyes, such as DAPI, were not sensitive enough to study the little amount of cytosolic DNA, so we utilized a specific antibody recognizing DNA. In the control cells almost all the stained dots in the cytoplasm were colocalized with mitochondria; while with BIX‐01294 treatment, stronger DNA signals not associated with mitochondria were observed and some dots were colocalized with histone H3 (Figure 1D). These indicated that BIX‐01294 increased the amount of cytosolic DNA, which might be translocated from nuclear and form chromatin‐like structure with histones. Antibodies recognizing histones or histone modifications were collected and applied in the immunostaining assay. Interestingly, we detected H4 and S10 phosphorylated H3 in cytoplasm, but not others (Figure 1E; Figure S2A‐C, Supporting Information). Since H3S10 phosphorylation is considered as one histone modification usually occurring inside of nuclear,^[^ ^19^ ^]^ the detected cytosolic histone was probably translocated from nuclear and not the newly‐synthesized histone. The reason we did not find other modified histones may be because only histone with certain modifications was selected to be translocated.

### 2.3 Heterochromatin Dysregulation Induces Cytosolic Histone H3

**Figure 2:**
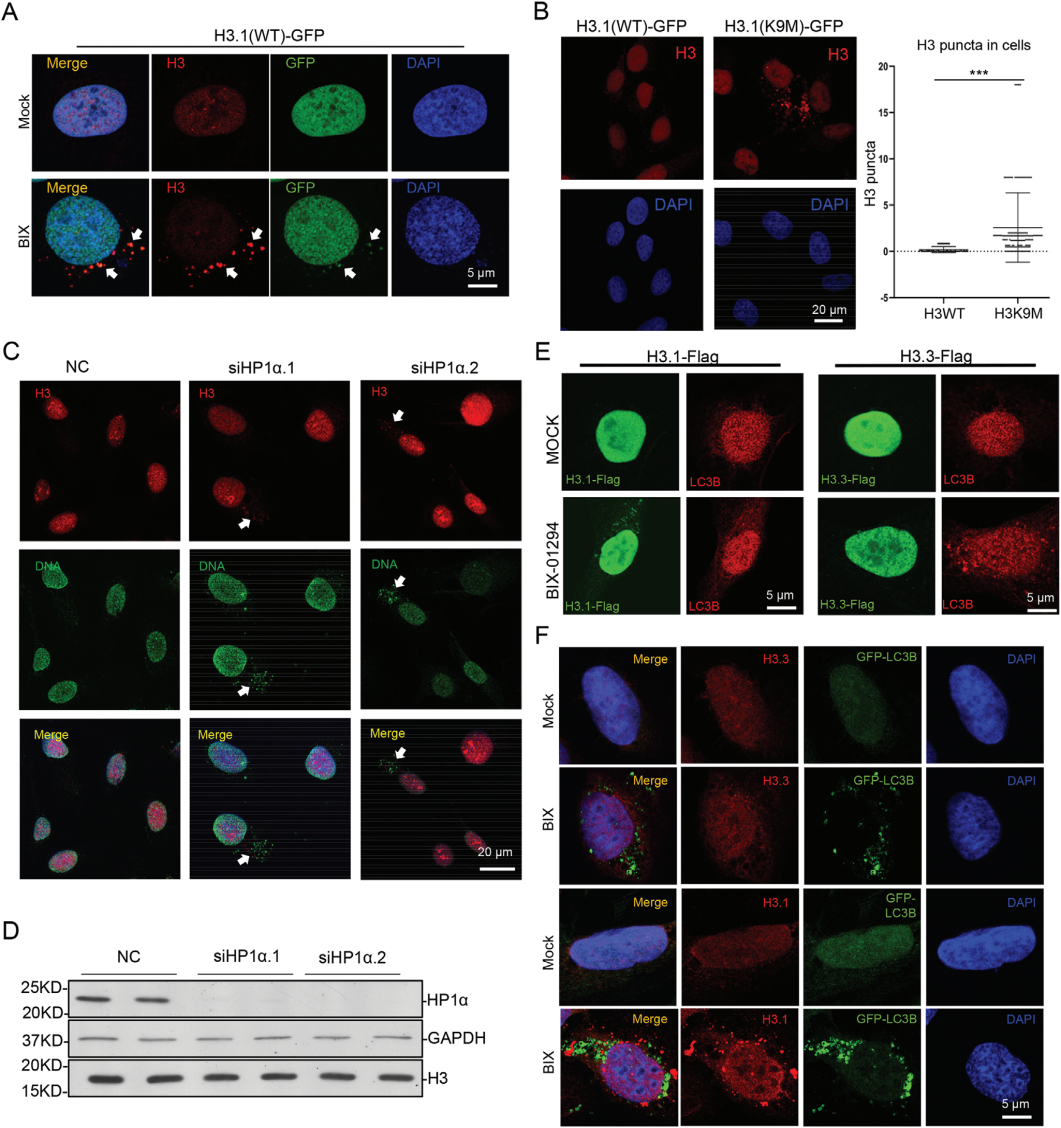
Dysregulation of heterochromatin induces cytosolic localization of histone H3.1. A) U2OS cells stably expressing H3.1‐GFP were treated with 5 µm BIX‐01294 for 8 h and imaged with confocal microscopy. Scale bar, 5 µm. B) U2OS cells stably expressing wild type or K9M H3.1‐GFP were imaged with confocal microscopy. Scale bar, 20 µm. The average number of cytosolic H3 puncta is shown at right (*n* (WT) = 121, *n* (K9M) = 151). Statistical analyses were performed with *t*‐test. ^***^ means *p* value < 0.001. C) *HP1α* was knocked down in U2OS cells with siRNAs. H3 and DNA were stained with antibodies and imaged with confocal microscopy. Scale bar, 20 µm. D) The cells used in (C) were analyzed with immunoblotting as indicated, H3 and GAPDH as loading controls. E) U2OS cells expressing Flag‐H3.1 or Flag‐H3.3 were treated with 5 µm BIX‐01294 for 8 h, and imaged with confocal microscopy. Scale bar, 5 µm. F) GFP‐LC3‐U2OS cells were treated with 5 µm BIX‐01294 for 8 h and stained with H3.1 or H3.3 antibodies, and imaged with confocal microscopy. Scale bar, 5 µm.

Since histone methylases often have non‐histone substrates, we used H3K9M mutant, which has been proved to repress global H3K9 methylation in cells,^[^ ^20^ ^]^ to investigate whether the modifications on histone H3 is responsible for H3 translocation. GFP‐H3.1 was first exogenously expressed in cells and BIX‐01294 was applied to induce cytosolic localization of GFP‐tagged H3 (**Figure** **2**A). We confirmed that H3.1‐K9M mutant could repress H3K9me3 in the cell (Figure S3A, Supporting Information), and further found that in H3.1K9M‐GFP stably expressed cells cytosolic H3 was observed without drug treatment (Figure 2B). Since SETDB1 catalyzes H3K9me3 on heterochromatin, we investigated whether heterochromatin dysregulation could cause the same phenotype. *HP1α* is one of the key proteins for heterochromatin structure.^[^ ^8^ ^]^ We knocked down *HP1α* with two different siRNAs and found that *HP1α* deficiency also triggered cytosolic H3 and DNA (Figure 2C,D). Interestingly, when two H3 variants, H3.1 and H3.3, were compared in the assay, only the exogenously expressed H3.1 was observed in the cytoplasm with BIX‐01294 treatment, but not H3.3 (Figure 2E). The specific antibodies for endogenous H3.1 and H3.3 were used in the study and the similar result was observed (Figure 2F). It is well documented that H3.1 tends to distribute on heterochromatin while H3.3 on actively transcribing genes.^[^ ^21^ ^]^ The above results together indicate that heterochromatin dysregulation selectively induces the translocation of nuclear materials from nucleus to cytoplasm. For convenience, we called the process CLIC, for cytoplasmic localization of nuclear material induced by chromatin dysregulation, in the following paragraphs.

### 2.4 Screen for Factors Involved in CLIC

To investigate whether other reagents can induce CLIC, we screened a series of epigenetic‐related chemicals, as well as rapamycin, an autophagy inducer, and etoposide, a DNA damage inducer. We found that only the inhibitors for H3K9 methylation could induce CLIC, including UNC0631 and UNC0638, but not others (Figure S3B,C, Supporting Information, and data not shown). To investigate whether CLIC occurs in other cell lines, several cell lines were tested and CLIC was observed in BJ, a primary cell line, HeLa, and HCT116 cancer cell lines (Figure S4, Supporting Information). These indicate that CLIC can occur in many cell lines and is tightly related with H3K9 methylation and heterochromatin.

To identify factors in the CLIC puncta, we tried to purify the complex. But probably because of its low amount, we failed to do so. Then we decided to perform an antibody screen based on immune‐staining. We got a test kid from Abclonal, which contained antibodies for around 50 selected nuclear proteins. Three proteins were identified to translocate into cytoplasm after BIX‐01294 treatment, including CCCTC‐binding factor (CTCF), MRE11, and high mobility group box 2 (HMGB2) (Figure S5, Supporting Information). Thus, together with H3 and H4, we totally identified five proteins existing in CLIC puncta. Besides these, SUN1 and SMC2 showed possible presentation in cytosol as well, which is worthy of further investigation.

### 2.5 CLIC is Different from Micronuclei

It is well known that micronuclei can be observed in some cells and induced during certain circumstance.^[^ ^22^ ^]^ We compared CLIC with micronuclei and found at least three differences exist between them, 1) micronuclei are packaged by lamina,^[^ ^23^ ^]^ but CLIC is not (Figure S6A, Supporting Information); 2) usually multiple puncta for CLIC are observed in one cell but only one or two for micronuclei (Figure S6A, Supporting Information); 3) the average diameter for micronuclei is at 2–3 µm, but that for CLIC is smaller than 1 µm (Figure S6B, Supporting Information). Based on these, we consider CLIC should have different structure and components from micronuclei.

### 2.6 CLIC is Different from Senescence‐Induced Nucleophagy

Degradation of lamin B1 (LMNB1) is one of the hallmarks for senescence‐associated nucleophagy.^[^ ^5^, ^24^ ^]^ We also observed the downregulation of endogenous and exogenously expressed lamin B1 after BIX‐01294 treatment, which was partially restored by the addition of Baf‐A1 (Figure S7A‐D, Supporting Information). The repression of lamin B1 by BIX‐01294 behaved in a dose dependent manner, but not obvious at low dose of 5 µm (Figure S7C, Supporting Information). In fact, BIX‐01294 also repressed the mRNA level of endogenous LMNB1 (Figure S7E, Supporting Information), which means BIX‐01294 repressed lamin B1 at both transcription and post‐translation level. Recent studies reported that during senescence lamin B1 enters cytoplasm and is degraded by autophagy.^[^ ^25^ ^]^ But we did not see any LMNB1 signal in CLIC puncta (Figure S6A, Supporting Information), indicating CLIC is different from their observation which showed positive lamin B1 signal in cytosol.

To further investigate the relationship between SETDB1‐mediated H3K9me3 and BIX‐01294‐induced process, H3K9me3 ChIP‐Seq was performed in SETDB1 knockout and BIX01294‐treated cells. H3K9me3 is mainly distributed in the intergenic regions and introns and BIX‐01294 greatly repressed its level, as expected (Figure S8A,B, Supporting Information). To study whether the process triggered by BIX‐01294 was similar to the senescence‐associated nucleophagy, we investigated the H3K9me3 level in the telomere regions. Interestingly, though H3K9me3 enrichment decreased on many chromatin loci in SETDB1 knockout cells, including telomeres, BIX‐01294 treatment acted differently on telomeres (Figure S8C,D, Supporting Information), suggesting telomeres were perhaps not involved in BIX‐01294‐dependent CLIC formation. Positive staining of *β*‐Gal is one of the hallmarks for senescent cells, however, BIX‐01294 treatment did not enhance senescence compared with the control cells (Figure S8E, Supporting Information). It was reported that H3K9 methylation increases in the senescent cells^[^ ^26^ ^]^ and our observation was consistent with the previous reports. Taken together, the data indicate that inhibition of H3K9me3 does not induce senescence. CLIC is probably a different pathway compared with senescence‐associated nucleophagy.

### 2.7 Down Regulation of H3K9me3 on SINE Transposons by SETDB1 Depletion and BIX‐01294 Treatment

**Figure 3:**
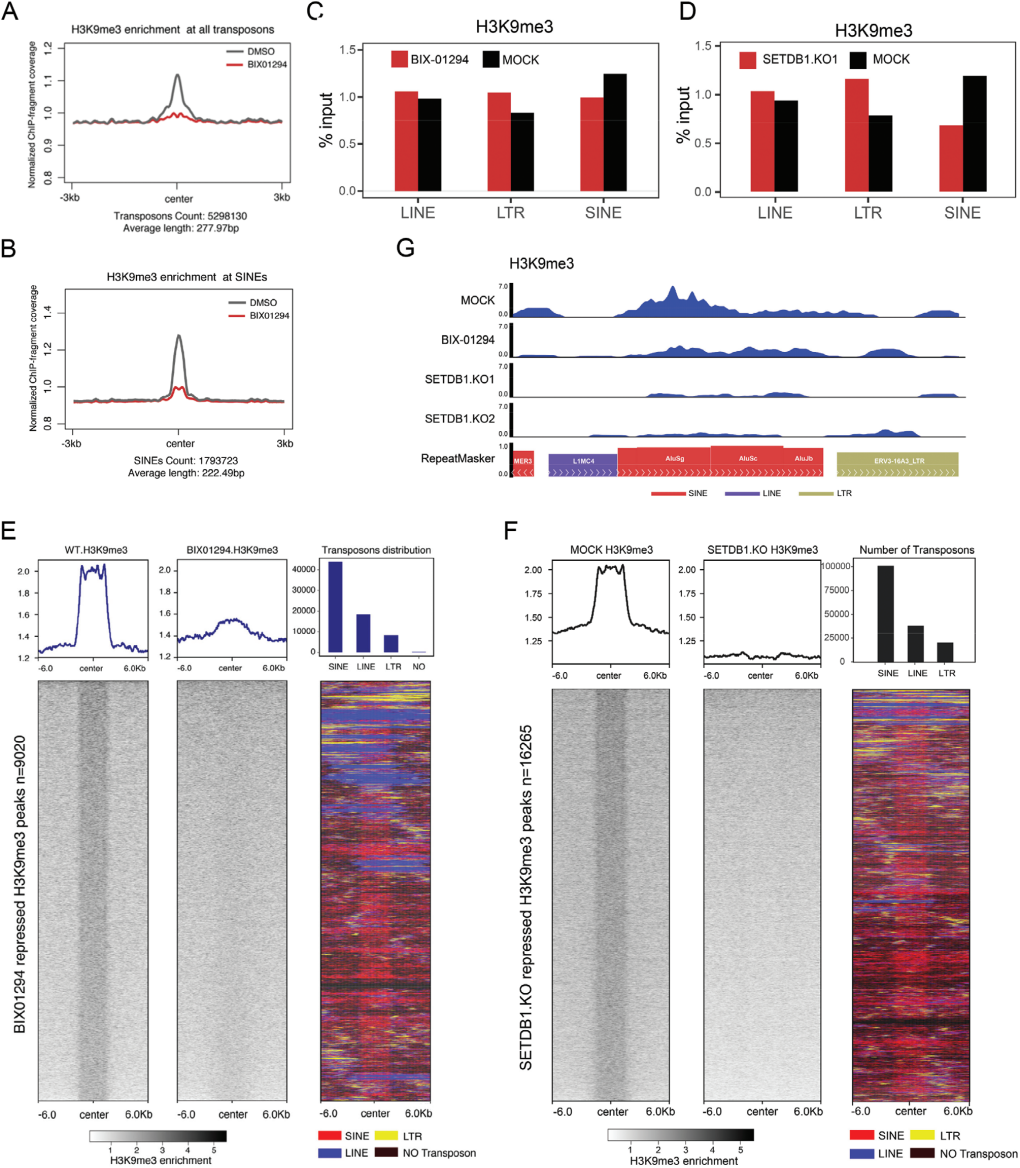
CLIC is probably associated with H3K9me3 down‐regulation on SINE transposons. A) U2OS cells were treated with 10 µm BIX‐01294 for 12 h. Metagene analysis of mean histone mark H3K9me3 ChIP‐Seq density is shown. The plot is centered at the retrotranspon element (RTE) regions. B) Metagene analysis shows the normalized average H3K9me3 enrichment on all SINEs decreased after BIX‐01294 treatment. C,D) Relative enrichment of H3K9me3 of RTE families in the control and BIX‐01294‐treated cells (C), or in the control and SETDB1 KO cells (D). *Y*‐axis represents the ratio of H3K9me3 tag number for each RTE family to the input tag number. E,F) Heat maps show the transposons (SINE, LINE, and LTR) distribution around the H3K9me3 peaks repressed by SETDB1 KO (E) or BIX‐01294 treatment (F). Each row represents one scaled H3K9me3 peak that includes ±6 kb of flanking regions. SINEs are labeled in red, LTRs in yellow, LINEs in blue, and regions without retrotranspons in black. The data indicate that SINEs were centered in the middle of H3K9me3 regions repressed by SETDB1 KO (E) or BIX‐01294 treatment (F). G) Genome browser view of H3K9me3 enrichment on one representative RTE region in the control, BIX‐01294‐treated and SETDB1 KO cells.

Previous studies showed that SETDB1 is responsible for catalyzing H3K9me3 on transposons and our ChIP‐Seq found that BIX‐01294 treatment also caused down‐regulation of H3K9me3 on transposons, especially on short interspersed nuclear elements (SINEs) (**Figure** **3**A,B). We analyzed the ratio of each type of transposons between ChIP‐Seq and input samples and found that BIX‐01294 treatment mainly decreased the number of H3K9me3 peaks on SINEs, which was similar to that in SETDB1 knockout cells (Figure 3C,D). The heat map of H3K9me3 peaks on all changed transposons showed that the down‐regulated H3K9me3 peaks in SETDB1 knockout cells or BIX‐01294‐treated cells were enriched mostly on SINEs, and less on long terminal repeats (LTRs) and long interspersed nuclear elements (LINEs) (Figure 3E,F). RNA‐seq results indicate that the number of up‐regulated SINEs was more than those down‐regulated (Figure S9A‐C, Supporting Information). These suggest the dysregulation of SINEs was perhaps related with CLIC induced by inhibition of H3K9 methylation. The genome browser view of one genomic locus enriched with SINEs is shown (Figure 3G). These results, together with the previous data, suggest that disruption of heterochromatin structure, especially on SINE regions, induces cytosolic localization of histone H3.1 and DNA.

It is reported previously increased retrotransposon expression could activate innate immunity and immune gene expression.^[^ ^27^ ^]^ Four expression plasmids containing non‐coding RNA 7SL, Alu element, SINE transposon B2, and transposon LINE1 were then transfected into U2OS cells respectively, but no signal for CLIC, or activated autophagy was observed (Figure S10A‐C, Supporting Information, and data not shown). The result suggests that CLIC is probably not induced by increased expression of retrotransposons.

### 2.8 Degradation of Cytosolic H3 by Autophagy

**Figure 4:**
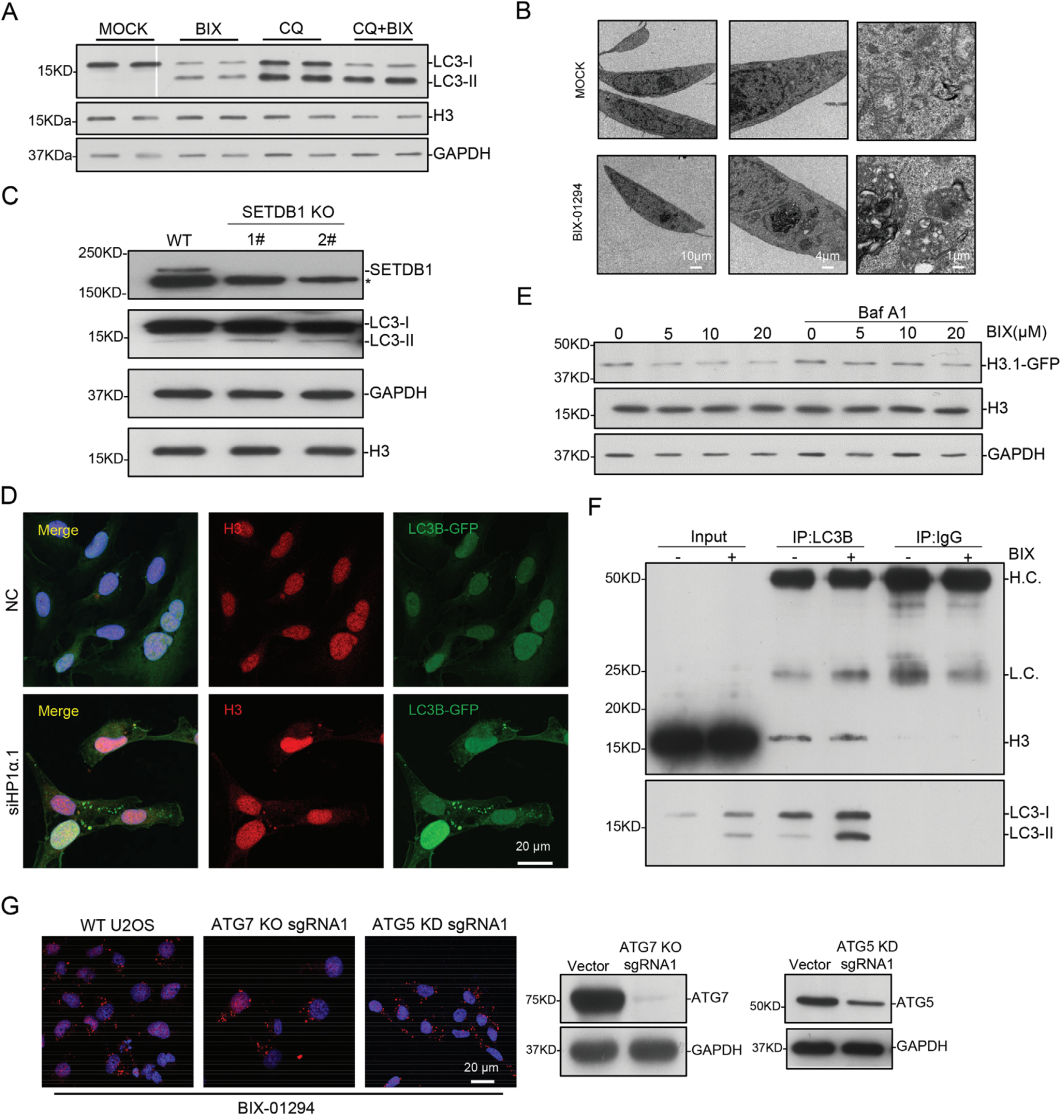
Cytosolic H3 is degraded by autophagy. A) U2OS cells were treated with 5 µm BIX‐01294 for 8 h w/wo 20 µm CQ for 24 h. LC3 level was analyzed with immunoblotting, GAPDH and H3 as loading control. B) U2OS cells were treated with 5 µm BIX‐01294 for 6 h and imaged with TEM. Scale bar, as indicated. C) WT and SETDB1‐KO U2OS cells were analyzed by immunoblotting with indicated antibodies, GAPDH, and H3 as control. ^*^ represents non‐specific bands. D) GFP‐LC3B‐U2OS cells were transfected with NC and *HP1α* siRNA, and imaged with confocal microscopy. Scale bar, 20 µm. E) U2OS cells stably expressing H3.1‐GFP were treated with the indicated concentrations of BIX‐01294 for 8 h with or without 100 nm Baf A1 for 24 h, and then analyzed with immunoblotting. F) Cells were treated with 5 µm BIX‐01294 for 12 h and the endogenous LC3 was immunoprecipitated. Western blotting was performed as indicated. HC, heavy chain. LC, light chain. G) WT, *ATG5* KD, and *ATG7* KO U2OS cells were treated with 5 µm BIX‐01294 for 8 h, and imaged with confocal microscopy. Scale bar, 20 µm. The level of *ATG5* and *ATG7* were analyzed with immunoblotting, GAPDH as loading control.

It is demonstrated that epigenetic chemical inhibitors induce autophagy in mammalian cells.^[^ ^14^, ^15^ ^]^ We repeated some experiments and observed that BIX‐01294 induces LC3II formation and autophagosomes in the cells (**Figure** **4**A,B). Increased amount of LC3II could be observed in SETDB1 knockout cells, and in a LC3‐GFP stable cell line, *HP1α* knockdown by siRNA induced LC3II aggregation, one hallmark for autophagy (Figure 4C,D). The previous immunostaining results have shown that a portion of histone H3 colocalized with LC3 in cytoplasm after BIX‐01294 treatment (Figure 1B). We made a movie of 3d image to show the co‐localization of H3 and LC3 (Movie S1, Supporting Information). Histone proteins are among the highest expressed proteins in mammalian cells and it is difficult to detect the change of endogenous H3. To investigate whether histone H3 in CLIC puncta can be degraded by autophagy, we utilized the GFP‐tagged H3.1 stable cell line. BIX‐01294 successfully decreased GFP‐H3.1 amount, which was rescued by Baf‐A1 addition (Figure 4E). Moreover, immunoprecipitation of LC3B pulled down histone H3 even without autophagy induction (Figure 4F), suggesting LC3 interacts with histone H3 in nucleus; and after BIX‐01294 treatment, LC3II helps H3 for degradation by autophagy in cytosol. While we failed to detect the interaction with bacteria‐purified proteins, suggesting the interaction is perhaps not direct. From the other side, autophagy deficiency by *autophagy related 7* *(ATG7)* knockout or *autophagy related 5* *(ATG5)* knockdown did not affect BIX‐01294‐induced CLIC puncta (Figure 4G). These results together indicated that the cytosolic H3.1 is degraded through autophagy process.

### 2.9 Activation of Inflammatory Genes by Cytosolic DNA

**Figure 5:**
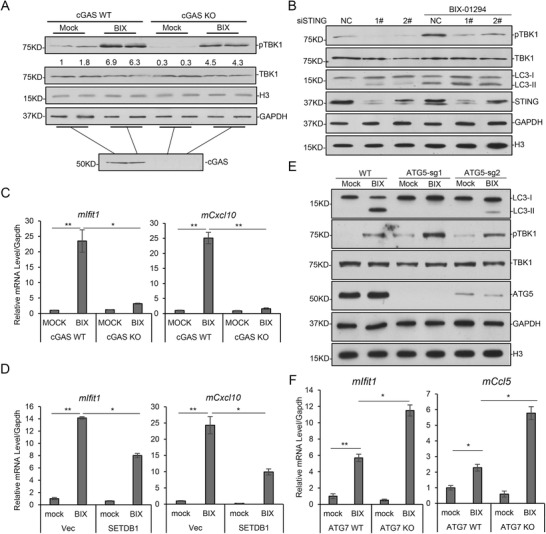
CLIC activates cGAS signal pathway. A) WT and cGAS KO L929 cells were treated with 10 µm BIX‐01294 for 12 h, and analyzed with immunoblotting as indicated, GAPDH and H3 as control. B) Sting was knocked down in L929 cells with siRNAs. Cells were then treated with 10 µm BIX‐01294 for 8 h and analyzed by immunoblotting, H3 and GAPDH as loading control. C) WT and cGAS KO L929 cells were treated with 10 µm BIX‐01294 for 12 h and the indicated genes were assayed with quantitative RT‐PCR. D) L929 cells expressing Flag‐SETDB1 were treated with 10 µm BIX‐01294 for 12 h and the indicated genes were assayed with quantitative RT‐PCR. E) *ATG5* was knocked down in U2OS with CRISPR. Then cells were treated with 10 µm BIX‐01294 for 8 h and analyzed by immunoblotting as indicated, GAPDH and H3 as loading control. F) WT and *ATG7* KO MEF cells were treated with 10 µm BIX‐01294 for 12 h and the indicated genes were assayed with quantitative RT‐PCR. Statistical analyses were performed with *t*‐test. ^**^ means *p* value < 0.01, ^*^ means *p* value < 0.05.

Previously we showed that cytosolic DNA increased after BIX‐01294 treatment (Figure 1D). cGAS (cyclic GMP‐AMP synthase) pathway is one important pathway for sensing cytosolic DNA, and the phosphorylation of TANK binding kinase 1 (TBK1) and expression of inflammatory genes are hallmarks for pathway activation.^[^ ^25^, ^28^ ^]^ Upon BIX‐01294 treatment, the phosphorylated TBK1 increased, and in a cGAS knockout L929 cell line, the induced Tbk1 phosphorylation decreased (**Figure** **5**A), suggesting the increased cytosolic DNA activated cGas/Tbk1 pathway. Knockdown of Sting/Mita showed the similar effect on BIX‐01294‐induced Tbk1 phosphorylation (Figure 5B). BIX‐01294 treatment also induced the expression of a series of inflammatory genes, which was repressed by cGas or Sting knockout, as well as by SETDB1 exogenous expression (Figure 5C,D; Figure S11A‐C, Table S1,S2, Supporting Information). Our ChIP‐Seq data supported that almost no H3K9me3 exists on these genes and their expression are not regulated by H3K9me3 in these cells (Figure S11D, Supporting Information). These indicated that the induced expression of these genes is not dependent on H3K9 methylation, but probably through cGas/Tbk1 pathway. Since cGAS is a sensor for DNA, cytosolic DNA induced during CLIC probably plays important roles in the expression of inflammatory genes.

### 2.10 Autophagy Blockage Enhanced the Activation of Inflammatory Genes

To investigate the role of autophagy in CLIC, we generated *ATG5* KD cell lines in U2OS with CRISPR technique (Figure 5E). *ATG5* deficiency inhibits BIX01294‐induced LC3II accumulation, indicating the autophagy process of LC3I to LC3II transformation is the same as the classical autophagy (Figure 5E). Interestingly, *ATG5* deficiency increased TBK1 phosphorylation (Figure 5E), and the induced expression of inflammatory genes was enhanced in *ATG7* ^−/−^ and *ATG5* deficient U2OS cells (Figure 5F; Figure S12A,B, Table S3, Supporting Information), suggesting blocking autophagy probably caused accumulated nuclear DNA in cytoplasm and enhanced cGAS pathway activation. Interestingly, BIX‐01294 treatment activated the expression of autophagy genes, such as *p62/SQSTM1* and LC3B, which were repressed by *ATG5* ablation (Figure S12C, Supporting Information). On the other hand, cGAS knockout did not affect the cytosolic localization of histone H3 and LC3II accumulation (Figure S12D,E, Supporting Information), indicating the activation of cGAS pathway is at the downstream of DNA translocation. The above results together suggest that autophagy induced by CLIC does not affect the translocation of nuclear materials, but promotes the degradation of nuclear materials in cytosol and thus restricted the activation of cGAS pathway.

### 2.11 Inhibition of H3K9 Methylation Induces Autophagy‐Dependent H2AX Phosphorylation

During the antibody screen we found that BIX‐01294 treatment induced the formation of *γ*H2AX foci in nuclei, especially with high dose and longtime treatment (Figure S13A, Supporting Information). Western blotting confirmed increased *γ*H2AX level in the cells (Figure S13B, Supporting Information), together with elevated level of phosphorylated ATM (Figure S13B, Supporting Information). Exogenous expression of SETDB1 inhibited the elevation of *γ*H2AX, indicating the involvement of histone methylation in the process (Figure S13C, Supporting Information). To compare CLIC with DNA damage response (DDR), we photographed the cells with BIX‐01294 or etoposide treatment. Etoposide induces large amount of *γ*H2AX in nuclei with 8 h treatment (Figure S13D, Supporting Information), but a high dose of BIX‐01294 (20 µm) induced much lower *γ*H2AX signal (Figure S13D‐F, Supporting Information). BIX‐01294‐induced cytosolic H3 could be observed after 1 h treatment (Figure S13D, Supporting Information), but no cytosolic H3 signal was observed for etoposide even when high *γ*H2AX signal was observed (Figure S13D, Supporting Information). These results indicated CLIC and DDR are different processes. Moreover, BIX‐01294 did not induce obvious activation of p53, even at high dose and longtime treatment (Figure S13E,F, Supporting Information). p53 activation is one of the hall marks of DDR, further suggesting CLIC is different from DDR.

Co‐knockdown of ATM serine/threonine kinase (ATM) and ATR serine/threonine kinase (ATR) reduced *γ*H2AX level induced by BIX‐01294 or UNC0638, suggesting the kinase for *γ*H2AX in CLIC is same as that in DDR (Figure S14A, Supporting Information). Then we checked *γ*H2AX level in *ATG7* knockout cells or with chloroquine (CQ) treatment, and found that autophagy inhibition repressed *γ*H2AX level (Figure S4B,C, Supporting Information), indicating autophagy is involved in *γ*H2AX induction.

### 2.12 *MRE11* is Involved in CLIC

**Figure 6:**
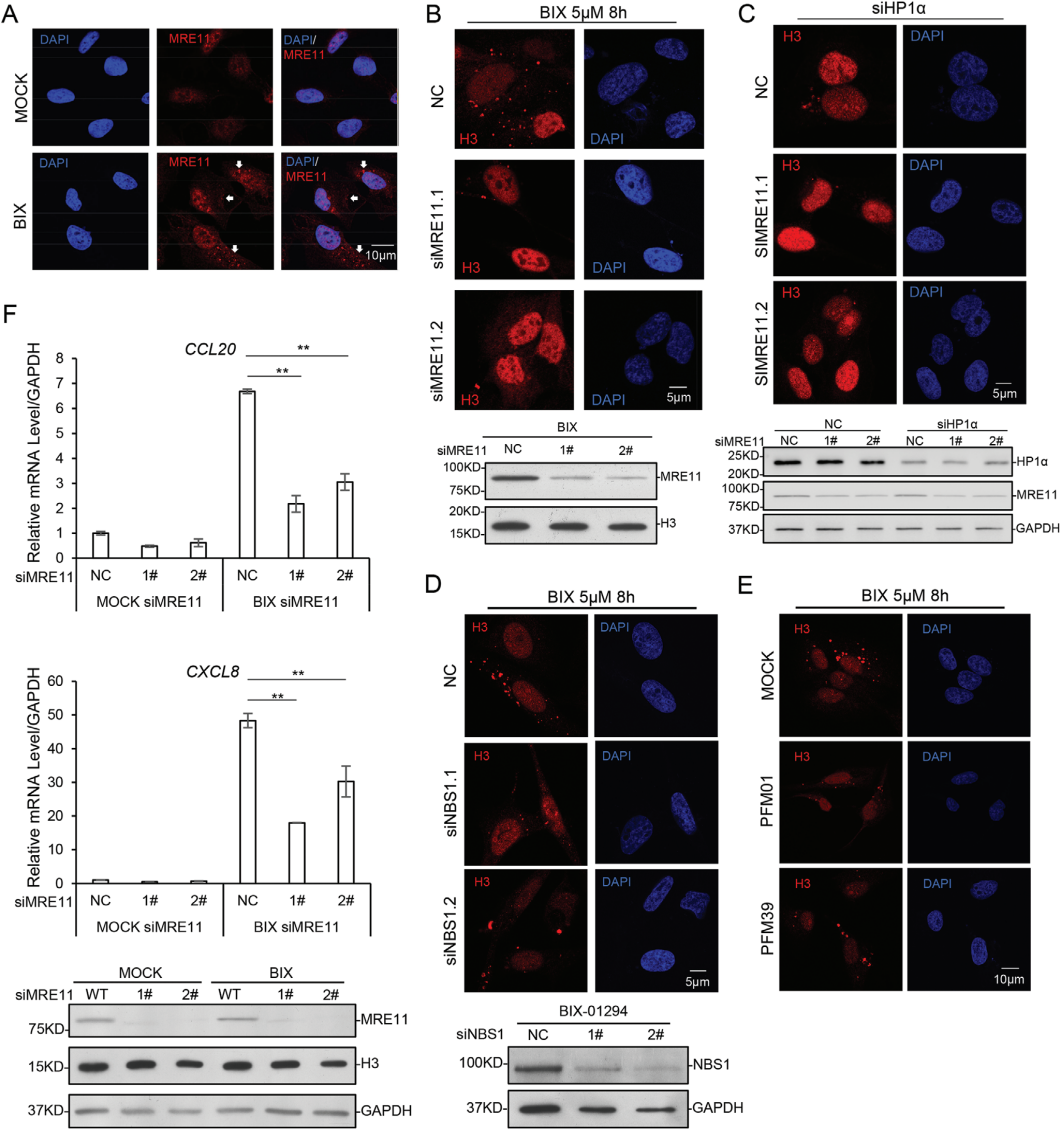
MRE11 and NBS1 are required for CLIC. A) U2OS cells were treated with 5 µm BIX‐01294 for 8 h, and imaged with confocal microscopy as indicated. The arrows indicate cytosolic MRE11 puncta. Scale bar, 10 µm. B) *MRE11* were knocked down in U2OS cells with siRNAs. Cells were treated with 5 µm BIX‐01294 for 8 h and analyzed with confocal microscopy. Scale bar, 5 µm. MRE11 level was analyzed by immunoblotting, H3 as loading control. C) *HP1α* was knocked down in U2OS or together with *MRE11*. Cells were then imaged with confocal microscopy. Scale bar, 5 µm. MRE11 and HP1α levels were measured by immunoblotting, GAPDH as loading control. D) *NBS1* was knocked down in U2OS cells. Cells were treated with 5 µm BIX‐01294 for 8 h and analyzed with confocal microscopy. Scale bar, 5 µm. NBS1 level was measured by immunoblotting, GAPDH as loading control. E) U2OS cells were treated with 5 µm BIX‐01294 for 8 h and w/wo 100 µm MRE11 inhibitors for 9 h (PFM01 for endonuclease activity, PFM39 for exonuclease activity), and imaged with confocal microscopy. Scale bar, 10 µm. F) *MRE11* was knocked down in U2OS. Cells were treated with 10 µm BIX for 12 h and the indicated genes were assayed with quantitative RT‐PCR. Statistical analyses were performed with *t*‐test. ^**^ means *p* value < 0.01. *MRE11* level was measured by immunoblotting, H3 and GAPDH as loading control.

Antibody screen showed that *MRE11*, a protein involved in DDR pathway, showed cytosolic distribution similar to H3 after BIX‐01294 treatment (**Figure** **6**A; Figure S5, Supporting Information). When *MRE11* was knocked down with siRNAs, the average number of CLIC puncta in each cell was significantly reduced (Figure 6B; Figure S15A, Supporting Information). To confirm the results, *HP1α* was knocked down to induce CLIC, and the co‐knockdown of *MRE11* greatly reduced the number of CLIC puncta in cells (Figure 6C; Figure S15B, Supporting Information). During DDR, MRE11 usually functions in the MRN complex with two subunits, RAD50 double strand break repair protein ( RAD50) and nibrin (NBN/NBS1).^[^ ^29^ ^]^ *NBS1* knockdown in cells also inhibits CLIC (Figure 6D; Figure S15C, Supporting Information). Interestingly, when *RAD50* was knocked down, the average number of CLIC puncta had no significant change (Figure S15D, Supporting Information). Meanwhile p53 activation by etoposide was repressed (Figure S15E, Supporting Information), indicating *RAD50* knockdown impaired DDR but not CLIC. The exogenously expressed *MRE11* in the above *MRE11* deficient cells successfully rescued the phenotype, while the *MRE11* mutants H129N, N117S, and R633Z could not (Figure S16A‐E, Supporting Information). H129N is a mutant defective in *NBS1* interaction, N117S for *MRE11* nuclease activities, and R633Z for DNA binding.^[^ ^30^ ^]^ These suggest that *NBS1* interaction, the nuclease activities and DNA binding are all required for *MRE11* function in CLIC. We further used PFM01 and PFM39, known inhibitors for *MRE11* endonuclease and exonuclease activities, respectively, to treat cells. Both inhibitors showed effects in inhibiting CLIC (Figure 6E; Figure S16F, Supporting Information), indicating the two nuclease domains of *MRE11* both contribute to CLIC puncta formation. Taken together, these results indicated that *MRE11* and *NBS1*, two subunits of MRN complex, is involved in the regulation of CLIC, independent of *RAD50*.

### 2.13 CLIC is Involved in Tumorigenesis

**Figure 7:**
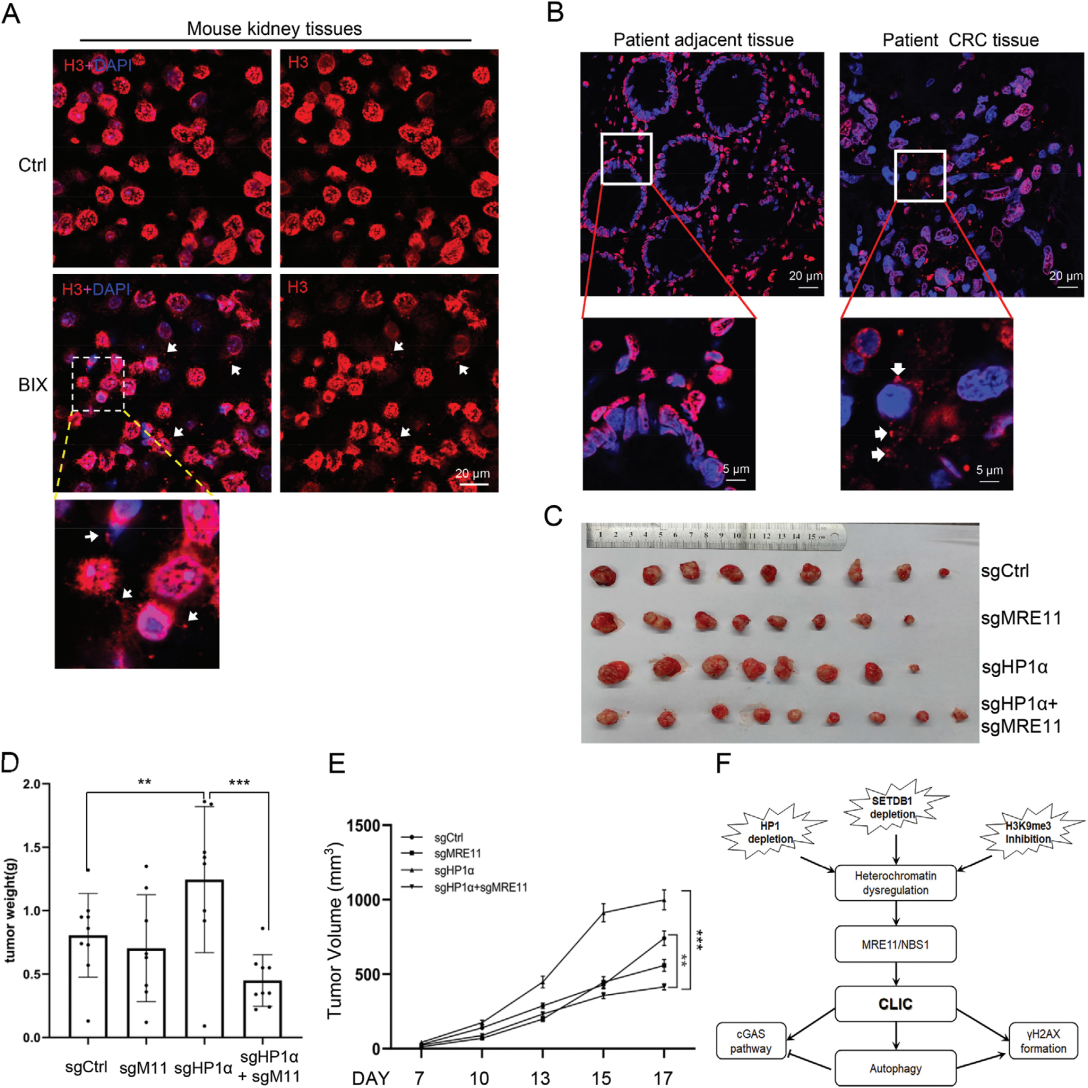
CLIC is associated with colorectal cancer. A) Mice were intraperitoneally injected with 20 mg kg^−1^ BIX‐01294 for 24 h. Representative H3 immunofluorescence images of kidney from treated and control mice is shown. The arrows indicate cytosolic CLIC puncta. Scale bar, 20 µm. B) Patient colorectal cancer tissue chips were immuno‐stained with H3 antibody and analyzed with confocal microscopy. Totally 8 cases of normal and 40 cases of cancer tissues were analyzed. Cytosolic H3 were found in 10% (4/40) cases of cancer tissues, but not in normal tissues. C–E) Xenograft experiments of *HP1α* and *MRE11* knockdown HCT116 colon cancer cells. 8 × 10^5^ cells were injected subcutaneously into the nude mice and tumors were harvested 18 days later. Tumors were pictured (C) and their weight (D) and volume (E) are shown as mean ± SEM, *n* (sgCtrl) = 9, *n* (sg*MRE11*) = 8, *n* (sgHP1*α*) = 8, and *n* (sgHP1*α* + sg*MRE11*) = 9. Statistical analyses were performed with *t*‐test. ^**^ means *p* value < 0.01, ^***^ for *p* value < 0.001. F) A sketch map for the processes associated with CLIC.

Based on the above results, we speculated CLIC might be related with inflammation and cancer. To investigate its potential biological significance, we first studied whether CLIC occurs in vivo. We injected BIX‐01294 into mice intraperitoneally and studied H3 level in multiple organs with immunostaining. We found H3 puncta in the cytosol increased in the BIX‐01294‐treated tissues, including kidney, liver, and intestine (**Figure** **7**A). Then we used the commercial tumor tissue chips from CRC patients, and performed immunostaining with H3 antibody. The adjacent native tissues were from the same patients, and the paired tissues were on the same chip to assure they had the same experimental condition. In around 10% of tumor tissues (4 out of 40 samples), more H3 puncta outside of nuclear were clearly observed compared with their paired adjacent tissues, suggesting CLIC occurs in a portion of CRC tumor cells (Figure 7B). To confirm the result, we generated *MRE11* knockdown, *HP1α* knockdown, and double knockdown cell lines with CRISPR/Cas9 system from HCT116 (Figure S16G, Supporting Information). We then performed xenograft experiments by injecting them into nude mice. The results showed that *MRE11* knockdown did not affect tumor growth significantly, while *HP1α* knockdown greatly enhanced it, indicating *HP1α* acts as a tumor suppressor in CRC. Co‐knockdown of *MRE11* and *HP1α* greatly decreased tumor growth compared with *HP1a* knockdown (Figure 7C–E; Figure S16H, Supporting Information), suggesting CLIC probably promotes tumor formation, which was induced by *HP1α* knockdown and then inhibited by *MRE11* knockdown.

## 3 Discussion

Cytosolic DNA has been shown to be associated with autoimmune diseases and cancers,^[^ ^1^ ^]^ however, what exactly happens on chromatin and how cytosolic DNA is generated remain elusive. Heterochromatin plays important roles in many aspects of cellular processes; however, it is still largely not known what the consequence is if its structure is impaired. Here, we show that defects in SETDB1‐mediated H3K9me3 on heterochromatin induces cytosolic localization of nuclear materials, including DNA, histone H3, H4, *MRE11*, HMGB2, and CTCF, which is called as CLIC. *MRE11* and *NBS1* are involved in the formation of CLIC puncta in cytoplasm. CLIC induction activates autophagy and cGAS/STING pathway in cells and is associated with tumorigenesis (Figure 7F).

In comparison with micronuclei, CLIC puncta do not have lamin B1 and many other proteins, and their size is much smaller than micronuclei, indicating CLIC puncta have different structure and components. CLIC is also different from classical DDR. CLIC appears in cytosol 1 h after BIX‐01294 treatment, while the weak *γ*H2AX signal appears much later. *RAD50* defect does not affect CLIC puncta formation, and inhibition of H3K9 methylation did not activate p53 obviously. These results indicate that CLIC is a novel process different from the known phenomena and pathways. Our discovery has revealed a novel process connecting epigenetic dysregulation with genome stability, autophagy, and inflammation, illustrated a novel pathway for the degradation of nuclear materials, and may contribute to the mechanistic study how nuclear DNA is transported into cytosol and causes diseases.

H3K9me3 down‐regulation often occurs in human diseases, such as cancer and diabetes,^[^ ^10^, ^31^ ^]^ and SETDB1 knockout in mice has been shown defects in development, tumorigenesis, and inflammatory response.^[^ ^7^, ^32^ ^]^ Previously scientists usually explained the underlying mechanisms through transcriptional regulation. Our study indicates that defects in SETDB1 or heterochromatin may cause severe consequences through transporting nuclear materials into cytosol. The cytosolic nuclear materials then activate cGAS pathway, which leads to expression of inflammatory genes and causes inflammation and other disorders.

Formation of *γ*H2AX foci is one of the hall marks for DDR. *γ*H2AX induced by CLIC is also mediated by ATM, showing similarity to DDR. It is not surprising since DNA is translocated into cytoplasm which is probably resulted from DNA cleavage from genomic DNA. In DDR, *γ*H2AX appears soon after DNA damage, while it is a much later event in CLIC. Meanwhile, autophagy inhibition significantly reduced *γ*H2AX level, suggesting a mechanism different from classical DDR exists. It was reported that virus infection induces *γ*H2AX elevation different from DDR.^[^ ^33^ ^]^ It is possible that chromatin dysregulation induces CLIC, and then unstructured chromatin is cut and transported outside of nuclear to be degraded by autophagy.

MRN complex formed by *MRE11*, *NBS1*, and *RAD50* has critical functions in DDR.^[^ ^29^, ^34^ ^]^ Our data show that *MRE11* can be translocated into cytosol, and knockdown of *MRE11* or *NBS1* reduces the number of CLIC puncta. Interestingly, *RAD50* does not have the above function. These indicate that *MRE11* and *NBS1* has *RAD50*‐independent function in CLIC, and regulation of CLIC shares some similar molecules as DDR pathway.

Based on our discoveries about CLIC, we speculated that it is perhaps related with cancer, inflammation, and auto‐immune diseases. We successfully observed CLIC puncta in BIX‐01294‐treated mice and CRC patient tissues. However, since small possibility may exist that careless sample preparation could cause false positive signals, future studies are important to verify our discoveries. Moreover, xenograft experiments showed that *MRE11* knockdown alone did not promote tumor growth, which was consistent with previous report;^[^ ^35^ ^]^ while co‐knockdown of *MRE11* and *HP1α* significantly repressed tumor growth caused by *HP1α* knockdown (Figure 7C–E). Considering the roles of *HP1α* and *MRE11* in CLIC, the repressed tumor formation was probably related with CLIC process, which strongly supports a connection between CLIC and tumorigenesis.

Besides these, many questions need to be solved by future studies. For example, how is the demethylated chromatin sensed? How is DNA cut and transported into cytosol with other nuclear materials? The physiological significance of CLIC also needs to be determined in animal models and more patient samples. To sum up, our study reveals a novel pathway by epigenetic dysregulation, which may play important roles in the maintenance of genome stability, inflammatory response, and other diseases.

## 4 Experimental Section

### Cell Lines and Tissue Culture

HEK293T, U2OS, L929, HeLa, and HCT116 cell lines were purchased from the Cell Bank of Chinese Academy. The primary cell lines BJ (RRID: CVCL_3653) and IMR90 (RRID: CVCL_0347) were from ATCC. MEF was isolated from mouse embryos in the lab. U2OS cells were cultured in RPMI 1640 supplemented with 10% FBS, 1% penicillin, and streptomycin. All the other cells were cultured in DMEM supplemented with 10% FBS, 1% penicillin, and streptomycin. All the cells were cultured at 37 °C with 5% CO_2_. U2OS cells were transfected with Lipofectamine 2000 (Invitrogen), and HEK293T cells were transfected with calcium phosphate.

### Antibodies and Reagents

Monoclonal antibodies against LC3B (Sigma, L7543, RRID: AB_796155), Flag (Sigma, F1804, RRID: AB_262044), HA (Origene, TA150034), EGFP (ABclonal, AE011, RRID: AB_2771922), *ATG5* (CST, 12,994, RRID: AB_2630393), *ATG7* (CST, 8558, RRID: AB_10831194), LMNB1 (Abcam, ab16048, RRID: AB_443298), H3K9me2 (Abcam, ab1220, RRID: AB_449854), GAPDH (Abclonal, AC002, RRID: AB_2736879), H3 (Abcam, ab1791, RRID: AB_302613; Active Motif, 39163, RRID: AB_2614978; Abclonal, A2348, RRID: AB_2631273), monoclonal antibody anti‐DNA (Progen, 61014, RRID: AB_2750935), H3K9me3 (Abcam, ab176916, RRID: AB_2797591; CST, 13969, RRID: AB_2798355), H3.1(Abclonal, A7010, RRID: AB_2767566), H3.3 (Abclonal, A10880, RRID: AB_2758292), H3K27me3 (CST, 9733, RRID: AB_2616029), H4K20me3 (Abclonal, A2372, RRID: AB_2764332), H4 (Abclonal, A1131, RRID: AB_2758500; CST, 13919, RRID: AB_2798345), p‐H3S10 (CST, 9706, RRID: AB_331748; Abclonal, AP0639 RRID:AB_2771169), SETDB1 (CST, 93212, RRID: AB_2800200), G9A (Abclonal, A1247, RRID: AB_2759314), SUV39H1 (Abclonal, A3277, RRID: AB_2765020), TBK1 (CST, 3504, RRID: AB_2255663), p‐TBK1 (CST, 5483, RRID: AB_10693472), cGAS (CST, 31659, RRID: AB_2799008),H3K4me1 (Abclonal, A2355 RRID:AB_2764315), H3K4me3 (Abclonal, A2357 RRID:AB_2631278), Sting (CST, 13647 RRID:AB_2732796), *MRE11* (Abcam, ab208020 RRID:AB_2814655; Abclonal, A2559 RRID:AB_2764447), *NBS1* (Abclonal, A0783 RRID:AB_2757395), *RAD50* (Abclonal, A3869 RRID:AB_2863151), *HP1α* (Abclonal, A1098 RRID:AB_2758354), *γ*H2AX (Abcam, ab26350 RRID:AB_470861), ATM (CST, 2873 RRID:AB_2062659), pATM(S1981) (Abcam, ab81292 RRID:AB_1640207), ATR (CST, 2790 RRID:AB_2227860), pCHK1 (CST, 2344 RRID:AB_331488), CHK1 (Epitomics, 2865‐1), p21 (CST, 2947 RRID:AB_823586), p53 (Santa Cruz, sc‐126 RRID:AB_628082), CTCF (Abclonal, A13272 RRID:AB_2760124), EP300 (Abclonal, A13016 RRID:AB_2759863), CENPH (Abclonal, A8372 RRID:AB_2768850), MED1 (Abclonal, A1724 RRID:AB_2763774), TBP (Abclonal, A16436 RRID:AB_2772540), RAD51 (Abclonal, A6268 RRID:AB_2766874), 53BP1 (Abclonal, A5757 RRID:AB_2766511), HMGB2 (Abclonal, A2973 RRID:AB_2764785), MCM3 (Abclonal, A1060 RRID:AB_2758140), SUN1 (Abclonal, A16024 RRID:AB_2763461), SMC1A (Abclonal, A2240 RRID:AB_2764244), and SMC2 (Abclonal, A17867 RRID:AB_2772312)were purchased from the indicated manufacturers. BIX‐01294, UNC0631, UNC0638, 2‐PCPA, and Baf A1 were purchased from Selleck. CQ, Rapamycin, and Etoposide were from Sigma Aldrich. DAPI, Alexa Flour 488, Alexa Flour 594 Secondary Antibody, and DAPI were from Thermo Fisher.

### Plasmids

pHage‐N‐Flag‐H3.1, pHage‐N‐Flag‐H3.3, pHage‐N‐HA‐GFP‐H3.1, pHage‐HA‐GFP‐H3.1 K9M, pHage‐N‐HA‐C‐3XFlag‐LMNB1, pHage‐HA‐*MRE11*, and pRK‐HA‐cGAS were cloned in the lab. pRK‐HA‐SETDB1 and pMX‐3XFlag‐SETDB1 were gifted by Dr. Jie‐Kai Chen of Guangzhou Institute of Biomedicine and Health, CAS. pAluYa5‐neo‐TET, L1‐neo‐TET, p7SL‐neo‐TET, pB2‐neo‐TET, pHage‐Flag‐GFP‐SUV39H1, and MSCV‐Flag‐G9A were purchased from Addgene. All plasmids were verified by sequencing before experiments.

### Cell Line Construction and Lentiviral Infection

Stable cell lines were made by lentivirus infection. Sg‐SETDB1, sg‐*ATG5*, and sg‐*ATG7* plasmid were constructed using LentiCRISPRv2 and lentiGuide‐Puro. Lentiviral pHage constructs were transfected with packaging plasmids to HEK293T cells. Viral supernatant was filtered through a 0.45 µm filter, supplemented with 8 µg mL^−1^ polybrene, and mixed with digested recipient cells. The infected cells were selected with puromycin about one week.

### Western Blotting

Cells were lysed in SDS loading buffer containing 50 mm Tris pH 6.82, 4% SDS, 0.2% bromophenol blue, 10% glycerol, and 5% *β*‐mercaptoethanol. The lysates were heated at 95 °C for minutes, and then subjected to electrophoresis using SDS‐PAGE gels. After transferring to nitrocellulose membrane, 5% milk in TBST buffer was used to block the membrane at room temperature for 1 h. Primary antibodies and of HRP‐conjugated secondary antibodies were incubated with the blot for 1 h in 5% milk/TBST. The membrane was washed and photographed with ECL.

### Immunoprecipitation

Cultured cells were harvested and lysed in lysis buffer (20 mm HEPES pH 7.4, 10% glycerol, 0.35 m NaCl, 1 mm MgCl_2_, 0.5% triton X‐100, and 1 mm DTT) with proteinase inhibitors. After removing insoluble particles, the supernatant was incubated with protein G beads (GE Healthcare) and specific antibody at 4 °C for 4 h. The beads were spin down and washed three times with lysis buffer. SDS loading buffer was added to the beads and western blotting was performed.

### Immunofluorescent Staining

Cells were cultured on the cover slips and fixed with freezing methanol after washed twice in PBS. The cover slips were then washed three times by PBS and blocked in PBS with 1% BSA and 0.1% NP‐40 for 10 min. The cover slips were hybridized with first and second antibodies for 1 h, respectively. Then the slips were mounted with prolong anti‐fade kit (Invitrogen) and observed with confocal fluorescent microscopy.

### Transmission Electron Microscopy

Cells were fixed in 4% glutaraldehyde. After dehydration, ultrathin sections were prepared using a microtome, and collected on grids. Sections were stained with 1% uranyl acetate and/or lead citrate, and images obtained with a Tecnai G20 TWIN transmission electron microscope.

### 3D Image of H3 and LC3 Localization in BIX‐01294‐Treated Cells

GFP‐LC3B‐U2OS cells were treated with 5 µm of BIX‐01294 for 8 h and then stained with H3 antibody, and analyzed by 3D Ariyscan microscopy. GFP‐LC3 is shown in green color, and H3 in red. Some H3 and LC3 puncta was co‐localized in the cytoplasm or associated with each other. The 3D image is provided as Movie S1, Supporting Information.

### RNA Interference, Reverse Transcription, and Quantitative PCR

The indicated cells were transfected with siRNA and were scraped down and collected by centrifugation. Total RNA was extracted with RNA extraction kit (Aidlab) according to manufacturer's manual. Approximately 1 µg of total RNA was used for reverse transcription with a first strand cDNA synthesis kit (Toyobo). The amount of mRNA was assayed by quantitative PCR. GAPDH was used to normalize the amount of each sample. Assays were repeated at least three times. Data shown were average values ± SD of three representative experiments. The information of primers is included in the Table S4, Supporting Information.

### Cell Viability Assay

Cells were split at 5 × 10^3^ per well in 96‐well plates. After 24 h of culture, cells were treated with varying concentrations of drugs for 48 or 72 h. Following incubation of cells in each well with MTT (0.25 µg) for 4 h at 37 °C, the medium with the formazan sediment was dissolved in 50% DMF and 30% SDS (pH 4.7). The absorption was read at 570 nm.

### Senescence‐Associated *β*‐Gal Assay

*β*‐Galactosidase assays were performed using a cellular senescence assay kit (Beyotime, C0602), according to the manufacturer's protocol. Cells were incubated with *β*‐gal detection solution at 37 °C overnight, and then imaged under regular light microscopy.

### RNA‐Seq Analysis

RNA‐seq library was performed by using Illumina TruSeq library construction kit. Using 5 µg total RNA as initiation, and then prepared according to the manufacturer's instruction. mRNA‐seq libraries were sequenced using HiSeq2500 for 100 bp paired‐end sequencing. Quality control of mRNA‐seq data was performed using Fatsqc and low‐quality bases were trimmed. All RNA‐seq data were mapped to the human genome (hg19) by TopHat (version 2.1.1) and allow maximum two mismatch. The gene expression level was calculated by Cufflinks with default parameters and gene ontology analysis was performed using DAVID (https://david.ncifcrf.gov).

### ChIP‐Assays

Approximately 1 × 10^7^ cells were fixed with 1% formaldehyde and quenched by glycine. The cells were washed three times with PBS and then harvested in ChIP lysis buffer (50 mm Tris‐HCl, pH7.6, 1 mm CaCl_2_, and 0.2% Triton X‐100). DNA was digested to 150–300 bp by MNase (Sigma) before extensive centrifugation. Four volume of ChIP dilution buffer (20 mm Tris‐HCl, pH 8.0, 150 mm NaCl, 2 mm EDTA, 1% Triton X‐100, and 0.1% SDS) was added to the supernatant. The resulted lysate was then incubated with protein G beads and antibodies at 4 °C overnight. The beads were washed five times and DNA was eluted by Chip elution buffer (0.1 m NaHCO_3_, 1% SDS, and 20 µg mL^−1^ proteinase K). The elution was incubated at 65 °C overnight and DNA was extracted with DNA purification kit (TIANGEN). The purified DNA was assayed by quantitative PCR with Biorad MyIQ.

### ChIP‐Seq Analysis

Library was prepared using the sequencing library preparation kit from Vazyme (ND604) according to the manufacturer's protocol. DNA was prepared for end repair and "A" tailing, adaptor ligation, and library amplification. ChIP‐Seq libraries were sequenced on HiSeq 2500 for 150 bp paired end sequencing.

Quality control of ChIP‐Seq data was performed using Fastqc. Low quality bases and adaptor contamination were deleted. Raw reads were aligned to the human genome (hg19) with Bowtie2 (version 2.1.0), and only uniquely mapped reads retained. The redundant reads were removed using SAMtools. MACS2 (version 2.1.1) was used to call peaks. The genomic location of the peaks and their distance to the TSS of annotated genes were using the annotatePeaks.pl of HOMER.

### Xenograft Experiments in Mice

The 5‐week‐old male BALB/c nude mice were purchased from GemPharmatech Co.,Ltd. Colon cancer model was established by injecting subcutaneously 8 × 10^5^ HCT116 cells per site into the flank regions of the mice. Tumor volumes were measured two or three days once using calipers. Tumor volumes were calculated as *V* = 0.5 × length × width^2^. After 17 days of injection, the tumors were harvested and weighed.

### Quantitation of Cytosolic Puncta and Statistical Analysis

To count cytoplasmic H3 puncta, 30 vision fields (for the cells with drugs or gene knockdown or knockout, at least one cell with cytosolic H3 puncta in the field) were first randomly chosen, then the cell number and cytoplasmic H3 puncta number in all fields were counted and the average puncta number per cell was calculated. For cell based experimental studies, the assays were repeated at least with three biological replicates. The sample size of each experiment is shown in the corresponding figure or legend. Data are shown as average values ± SD or SEM. *p* value was calculated using Student's *t* test (two‐sided), and statistical significance was assigned with ^*^ *p* value < 0.05, ^**^ *p* value < 0.01, ^***^ *p* value < 0.001.

### Ethics Approval and Consent to Participate

All the animal operations were following the laboratory animal guidelines of Wuhan University and were approved by the Animal Experimentations Ethics Committee of Wuhan University (Protocol NO. 14110B). No patient study was involved and the consent to participate was not applicable.

## Author Contributions

Conceptualization, M.W.; experiment performing, Z.W., J.C., Q.X., X.‐W.W., and S.‐B.T.; bioinformatic analyses, C.G., Q.‐L.L.; providing critical reagents, H.‐B.S., Z.B.; manuscript writing, M.W., Z.W.; funding acquisition and supervision, M.W., L.‐Y.L.

## Conflict of Interest

The authors declare no conflict of interest.

## Supporting information

Supporting Information

Supporting Movie 1

Supporting Table 1

## Data Availability

The original data file for deep sequencing were submitted to GEO database with Acc. No. GSE140402.

## References

[1] M. M. Hu , H. B. Shu , Annu. Rev. Immunol. 2020, 38, 79. 31800327 10.1146/annurev-immunol-070119-115052

[2] L. Andreeva , B. Hiller , D. Kostrewa , C. Lassig , C. C. de Oliveira Mann , D. Jan Drexler , A. Maiser , M. Gaidt , H. Leonhardt , V. Hornung , K. P. Hopfner , Nature 2017, 549, 394. 28902841 10.1038/nature23890

[3] S. F. Bakhoum , B. Ngo , A. M. Laughney , J. A. Cavallo , C. J. Murphy , P. Ly , P. Shah , R. K. Sriram , T. B. K. Watkins , N. K. Taunk , M. Duran , C. Pauli , C. Shaw , K. Chadalavada , V. K. Rajasekhar , G. Genovese , S. Venkatesan , N. J. Birkbak , N. McGranahan , M. Lundquist , Q. LaPlant , J. H. Healey , O. Elemento , C. H. Chung , N. Y. Lee , M. Imielenski , G. Nanjangud , D. Pe'er , D. W. Cleveland , S. N. Powell , J. Lammerding , C. Swanton , L. C. Cantley , Nature 2018, 553, 467. 29342134 10.1038/nature25432 PMC5785464

[4] a) A. Ivanov , J. Pawlikowski , I. Manoharan , J. van Tuyn , D. M. Nelson , T. S. Rai , P. P. Shah , G. Hewitt , V. I. Korolchuk , J. F. Passos , H. Wu , S. L. Berger , P. D. Adams , J. Cell Biol. 2013, 202, 129; 23816621 10.1083/jcb.201212110 PMC3704985

[5] Z. Dou , C. Xu , G. Donahue , T. Shimi , J. A. Pan , J. Zhu , A. Ivanov , B. C. Capell , A. M. Drake , P. P. Shah , J. M. Catanzaro , M. D. Ricketts , T. Lamark , S. A. Adam , R. Marmorstein , W. X. Zong , T. Johansen , R. D. Goldman , P. D. Adams , S. L. Berger , Nature 2015, 527, 105. 26524528 10.1038/nature15548 PMC4824414

[6] a) D. Nicetto , K. S. Zaret , Curr. Opin. Genet. Dev. 2019, 55, 1; 31103921 10.1016/j.gde.2019.04.013 PMC6759373

[7] T. Matsui , D. Leung , H. Miyashita , I. A. Maksakova , H. Miyachi , H. Kimura , M. Tachibana , M. C. Lorincz , Y. Shinkai , Nature 2010, 464, 927. 20164836 10.1038/nature08858

[8] W. Zeng , A. R. Ball, Jr. , K. Yokomori , Epigenetics 2010, 5, 287. 20421743 10.4161/epi.5.4.11683 PMC3103764

[9] a) A. H. Peters , D. O'Carroll , H. Scherthan , K. Mechtler , S. Sauer , C. Schofer , K. Weipoltshammer , M. Pagani , M. Lachner , A. Kohlmaier , S. Opravil , M. Doyle , M. Sibilia , T. Jenuwein , Cell 2001, 107, 323; 11701123 10.1016/s0092-8674(01)00542-6

[10] Q. Y. Zhao , P. J. Lei , X. Zhang , J. Y. Zheng , H. Y. Wang , J. Zhao , Y. M. Li , M. Ye , L. Li , G. Wei , M. Wu , Clin. Epigenet. 2016, 8, 34. 10.1186/s13148-016-0201-x PMC4815258 27034728

[11] J. Fullgrabe , D. J. Klionsky , B. Joseph , Nat. Rev. Mol. Cell Biol. 2014, 15, 65. 24326622 10.1038/nrm3716

[12] a) S. H. Baek , K. I. Kim , Mol. Cell 2017, 65, 781; 28257699 10.1016/j.molcel.2016.12.027

[13] W. Wang , Q. Wang , D. Wan , Y. Sun , L. Wang , H. Chen , C. Liu , R. B. Petersen , J. Li , W. Xue , L. Zheng , K. Huang , Autophagy 2017, 13, 941. 28409999 10.1080/15548627.2017.1293768 PMC5446066

[14] Y. Kim , Y. S. Kim , D. E. Kim , J. S. Lee , J. H. Song , H. G. Kim , D. H. Cho , S. Y. Jeong , D. H. Jin , S. J. Jang , H. S. Seol , Y. A. Suh , S. J. Lee , C. S. Kim , J. Y. Koh , J. J. Hwang , Autophagy 2013, 9, 2126. 24322755 10.4161/auto.26308

[15] Z. Wang , Q. Y. Long , L. Chen , J. D. Fan , Z. N. Wang , L. Y. Li , M. Wu , X. Chen , Biochim. Biophys. Acta 2017, 1864, 2428. 10.1016/j.bbamcr.2017.08.005 28800922

[16] J. D. Fan , P. J. Lei , J. Y. Zheng , X. Wang , S. Li , H. Liu , Y. L. He , Z. N. Wang , G. Wei , X. Zhang , L. Y. Li , M. Wu , PLoS One 2015, 10, e0116782. 25562686 10.1371/journal.pone.0116782 PMC4285553

[17] A. Artal‐Martinez de Narvajas , T. S. Gomez , J. S. Zhang , A. O. Mann , Y. Taoda , J. A. Gorman , M. Herreros‐Villanueva , T. M. Gress , V. Ellenrieder , L. Bujanda , D. H. Kim , A. P. Kozikowski , A. Koenig , D. D. Billadeau , Mol. Cell. Biol. 2013, 33, 3983. 23918802 10.1128/MCB.00813-13 PMC3811684

[18] a) S. Kubicek , R. J. O'Sullivan , E. M. August , E. R. Hickey , Q. Zhang , M. L. Teodoro , S. Rea , K. Mechtler , J. A. Kowalski , C. A. Homon , T. A. Kelly , T. Jenuwein , Mol. Cell 2007, 25, 473; 17289593 10.1016/j.molcel.2007.01.017

[19] K. M. Johansen , J. Johansen , Chromosome Res. 2006, 14, 393. 16821135 10.1007/s10577-006-1063-4

[20] H. M. Herz , M. Morgan , X. Gao , J. Jackson , R. Rickels , S. K. Swanson , L. Florens , M. P. Washburn , J. C. Eissenberg , A. Shilatifard , Science 2014, 345, 1065. 25170156 10.1126/science.1255104 PMC4508193

[21] P. Grover , J. S. Asa , E. I. Campos , Annu. Rev. Genet. 2018, 52, 109. 30183406 10.1146/annurev-genet-120417-031547

[22] C. J. Ye , Z. Sharpe , S. Alemara , S. Mackenzie , G. Liu , B. Abdallah , S. Horne , S. Regan , H. H. Heng , Genes 2019, 10, 366. 10.3390/genes10050366 PMC6562739 31086101

[23] a) E. M. Hatch , A. H. Fischer , T. J. Deerinck , M. W. Hetzer , Cell 2013, 154, 47; 23827674 10.1016/j.cell.2013.06.007 PMC3749778

[24] a) A. Turan , L. Grosche , A. Krawczyk , P. Muhl‐Zurbes , C. Drassner , A. Duthorn , M. Kummer , M. Hasenberg , S. Voortmann , H. Jastrow , J. Dorrie , N. Schaft , M. Kraner , K. Dohner , B. Sodeik , A. Steinkasserer , C. S. Heilingloh , J. Cell Biol. 2019, 218, 508; 30587512 10.1083/jcb.201801151 PMC6363456

[25] Z. Dou , K. Ghosh , M. G. Vizioli , J. Zhu , P. Sen , K. J. Wangensteen , J. Simithy , Y. Lan , Y. Lin , Z. Zhou , B. C. Capell , C. Xu , M. Xu , J. E. Kieckhaefer , T. Jiang , M. Shoshkes‐Carmel , K. Tanim , G. N. Barber , J. T. Seykora , S. E. Millar , K. H. Kaestner , B. A. Garcia , P. D. Adams , S. L. Berger , Nature 2017, 550, 402. 28976970 10.1038/nature24050 PMC5850938

[26] a) M. Reimann , S. Lee , C. Loddenkemper , J. R. Dorr , V. Tabor , P. Aichele , H. Stein , B. Dorken , T. Jenuwein , C. A. Schmitt , Cancer Cell 2010, 17, 262; 20227040 10.1016/j.ccr.2009.12.043

[27] W. Sheng , M. W. LaFleur , T. H. Nguyen , S. Chen , A. Chakravarthy , J. R. Conway , Y. Li , H. Chen , H. Yang , P. H. Hsu , E. M. Van Allen , G. J. Freeman , D. D. De Carvalho , H. H. He , A. H. Sharpe , Y. Shi , Cell 2018, 174, 549. 29937226 10.1016/j.cell.2018.05.052 PMC6063761

[28] a) W. W. Luo , H. B. Shu , Cell Mol. Immunol. 2018, 15, 666; 29456253 10.1038/cmi.2016.51 PMC6123429

[29] T. T. Paull , Mol. Cell 2018, 71, 419. 30057197 10.1016/j.molcel.2018.06.033

[30] T. Kondo , J. Kobayashi , T. Saitoh , K. Maruyama , K. J. Ishii , G. N. Barber , K. Komatsu , S. Akira , T. Kawai , Proc. Natl. Acad. Sci. U. S. A. 2013, 110, 2969. 23388631 10.1073/pnas.1222694110 PMC3581880

[31] a) J. C. Black , A. L. Manning , C. Van Rechem , J. Kim , B. Ladd , J. Cho , C. M. Pineda , N. Murphy , D. L. Daniels , C. Montagna , P. W. Lewis , K. Glass , C. D. Allis , N. J. Dyson , G. Getz , J. R. Whetstine , Cell 2013, 154, 541; 23871696 10.1016/j.cell.2013.06.051 PMC3832053

[32] a) G. Wang , J. Long , Y. Gao , W. Zhang , F. Han , C. Xu , L. Sun , S. C. Yang , J. Lan , Z. Hou , Z. Cai , G. Jin , C. C. Hsu , Y. H. Wang , J. Hu , T. Y. Chen , H. Li , M. G. Lee , H. K. Lin , Nat. Cell Biol. 2019, 21, 214; 30692626 10.1038/s41556-018-0266-1 PMC6414065

[33] G. A. Shah , C. C. O'Shea , Cell 2015, 162, 987. 26317467 10.1016/j.cell.2015.07.058 PMC4681434

[34] L. Bian , Y. Meng , M. Zhang , D. Li , Mol. Cancer 2019, 18, 169. 31767017 10.1186/s12943-019-1100-5 PMC6878665

[35] M. Petroni , F. Sardina , P. Infante , A. Bartolazzi , E. Locatelli , F. Fabretti , S. Di Giulio , C. Capalbo , B. Cardinali , A. Coppa , A. Tessitore , V. Colicchia , M. Sahun Roncero , F. Belardinilli , L. Di Marcotullio , S. Soddu , M. Comes Franchini , E. Petricci , A. Gulino , G. Giannini , Cell Death Dis. 2018, 9, 895. 30166519 10.1038/s41419-018-0924-z PMC6117286

