# Supplementary material for: Epigenetic Dysregulation Induces Translocation of Histone H3 into Cytoplasm
Source: Adv Sci (Weinh). 2021 Aug 7;8(19):2100779. doi: 10.1002/advs.202100779 (PMC8498869; doi:10.1002/advs.202100779)
Supplement: Supplementary file 1 — Supporting Information [file ADVS-8-2100779-s003.pdf]

© 2021 Wiley-VCH GmbH

ADVANCED SCIENCE
Open Access

Supporting Information

for *Adv. Sci.*, DOI: 10.1002/advs.202100779

Epigenetic Dysregulation Induces Translocation of Histone H3 into Cytoplasm

*Zhen Wang, Ji Chen, Chuan Gao, Qiong Xiao, Xi-Wei Wang, Shan-Bo Tang, Qing-Lan Li, Bo Zhong, Zhi-Yin Song, Hong-Bing Shu, Lian-Yun Li, and Min Wu**

Sup. Fig. 1

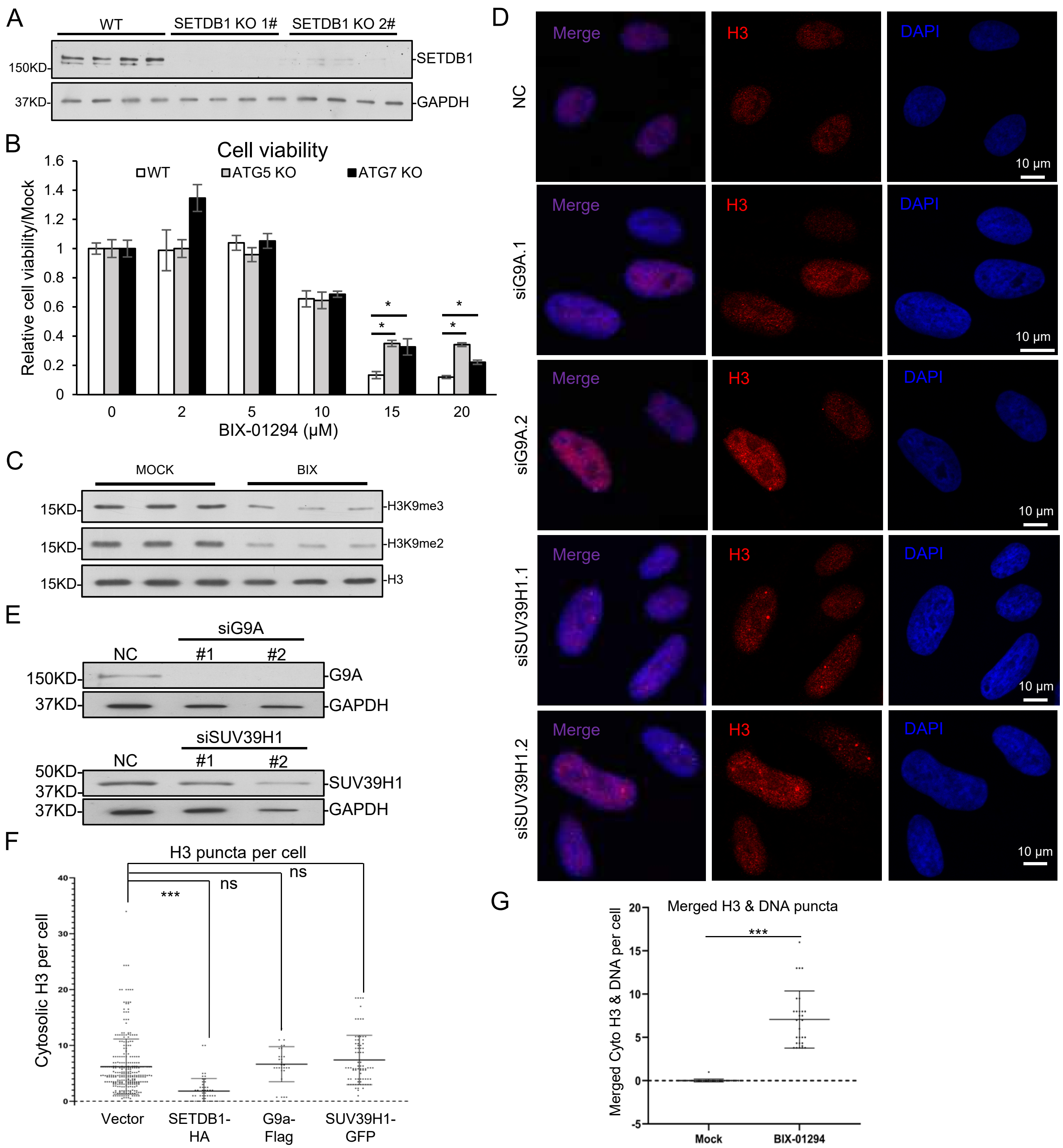

Sup. Fig. 1 G9a and SUV39H1 are not involved in the BIX-01294-induced H3 translocation. (A) SETDB1was knocked out in U2OS cells using CRISPR, and analyzed with immunoblotting, GAPDH as loading control. **(B)** WT, ATG5 KO and ATG7 KO U2OS cells were treated with the indicated concentrations of BIX-01294 for 48 hr, and MTT assay was performed. The ratios of survived cells relative to controls were shown. Statistical analyses were performed by t-test. **(C)** U2OS cell was treated with BIX-01294 at the indicated concentratins for 8 hr and analyzed with immunoblotting as indicated, H3 as loading control **(D&E)** G9A and SUV39H1 were knocked down with siRNAs in U2OS cells and analyzed with confocal microscopy. Scale bar, 10 μm. Immunoblotting of G9A and SUV39H1 in the cells was performed, GAPDH as loading control. **(F)** The number of cytosolic H3 puncta in Fig. 1C was counted manually, and the average number was shown (n (VEC) = 290, n (Flag-G9A) = 34, n (SUV39H1-GFP) = 79, n (HA-SETDB1) = 51). **(G)** The number of H3 and DNA merged puncta in Fig. 1D was counted manually, and the average numbers per cell were shown(n(Mock) = 36, n(BIX-01294) = 27).Statistical analyses were performed with t-test. *** means p value< 0.001.

Sup. Fig. 2

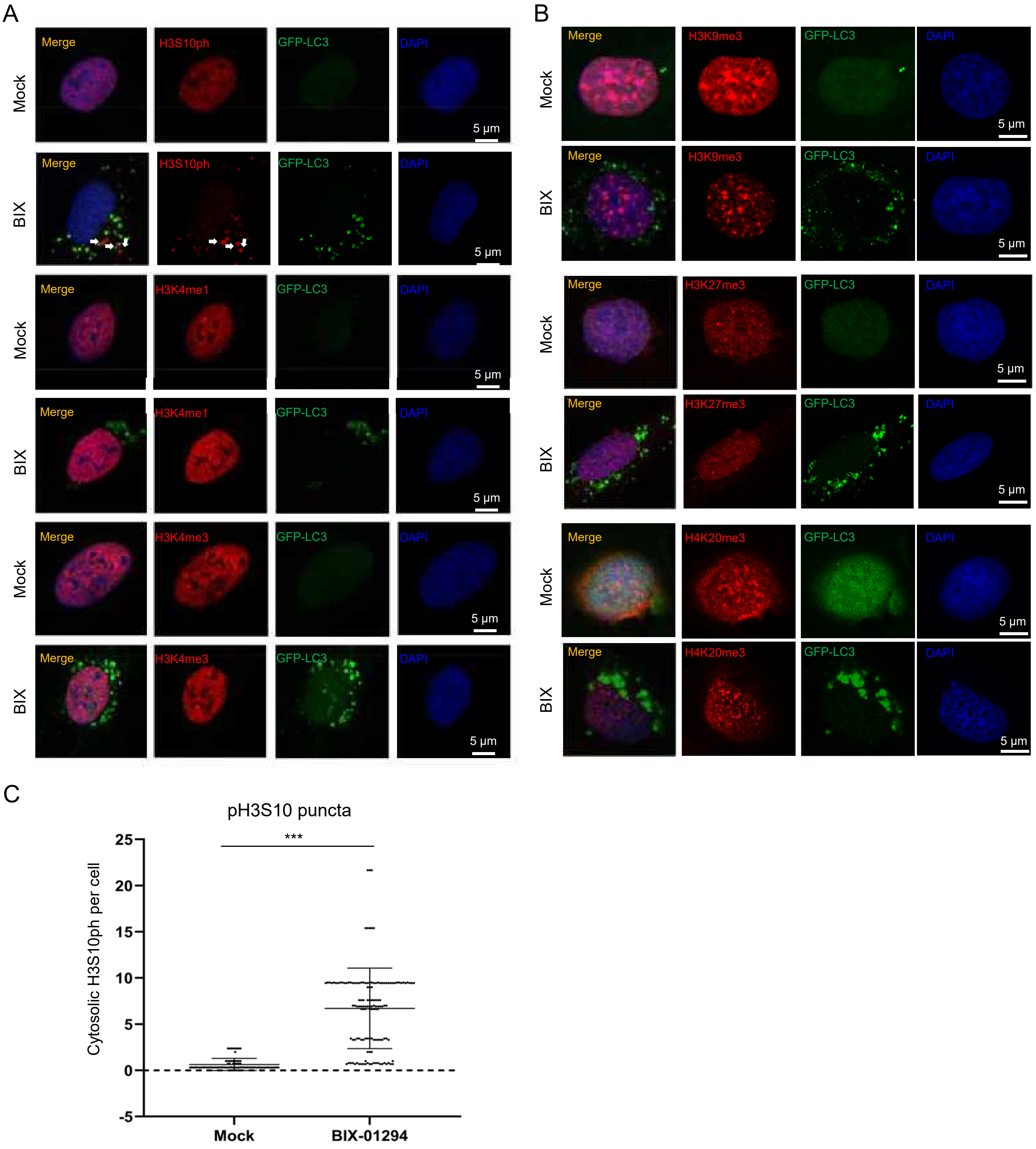

Sup. Fig. 2 Screen for modified histones in CLIC puncta. (A) GFP-LC3-U2OS cells were treated with 5 μM BIX-01294 for 8 hr and stained with pH3S10, H3K4me1, and H3K4me3 , and imaged with confocal microscopy. The white arrows show cytoplasmic puncta. Scale bar, 5μm. **(B)** GFP-LC3-U2OS cells were treated with 5 μM BIX-01294 for 8 hr and stained with H3K9me3, H3K27me3, and H4K20me3 , and imaged with confocal microscopy. Scale bar, 5μm. **(C)** The number of cytosolic H3S10ph puncta after BIX-01294 treatment in Sup. Fig. 3A was counted manually, and the average numbers were shown(n(Mock) = 80, n(BIX-01294) = 136). Statistical analyses were performed with t-test. *** means p value< 0.001.

Sup. Fig. 3

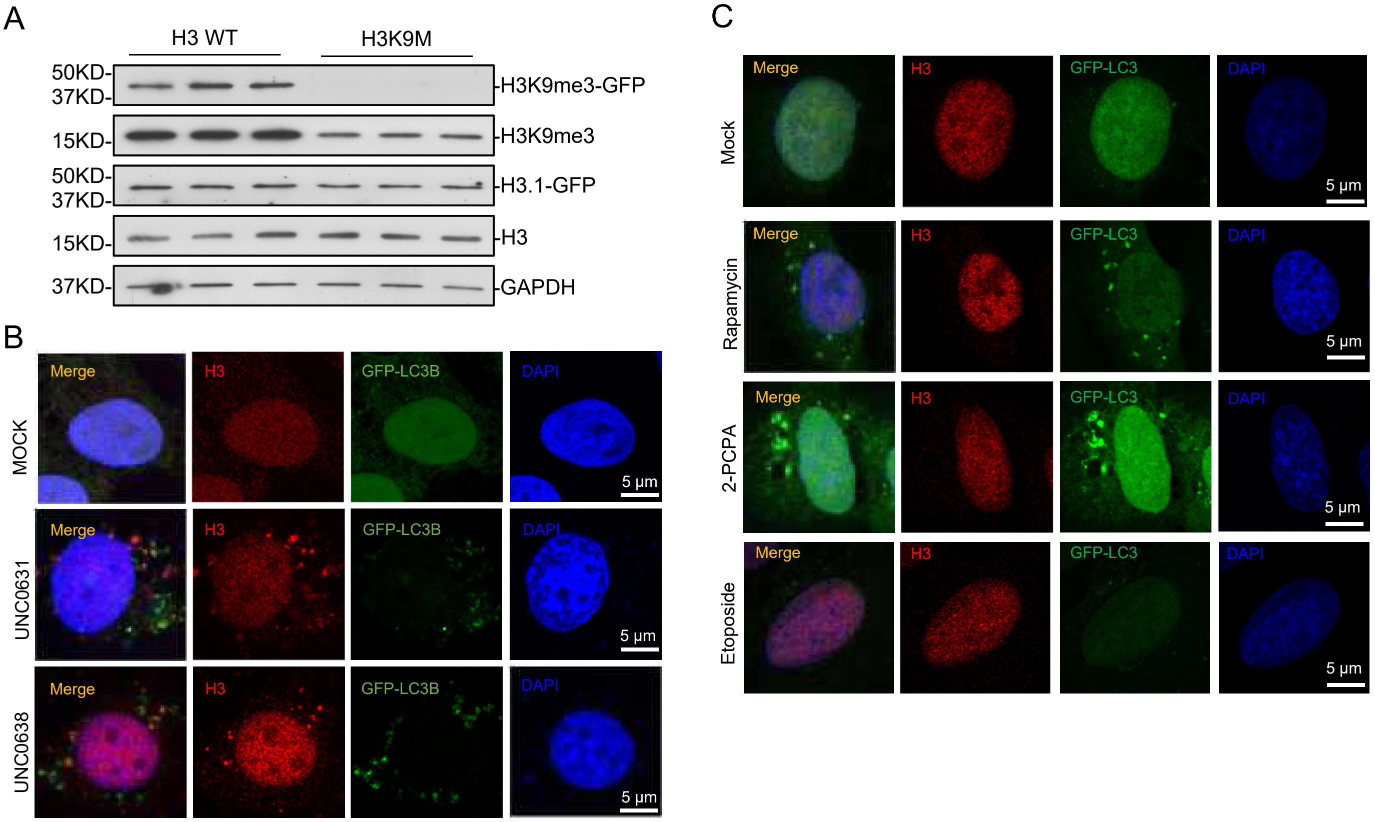

Sup. Fig. 3 Screen for small molecular chemicals able to induce CLIC. (A) U2OS cells expressing H3.1-GFP and H3.1 K9M-GFP were immunoblotted for H3K9me3, H3 and LC3, GAPDH as loading control. **(B)** Mock, UNC0631 and UNC0638-treated GFP-LC3-U2OS cells were analyzed by confocal microscopy. Scale bar, 5 µm. Cells were treated with 10 µM UNC0631 or 10µM UNC0638 for 8 hr. **(C)** GFP-LC3-U2OS cells were treated with 20 µM Rapamycin for 6 hr, 500 µM 2-PCPA for 12 hr or 50 µM etoposide for 8 hr, and analyzed by confocal microscopy, respectively. Scale bar, 5 µm.

Sup. Fig. 4

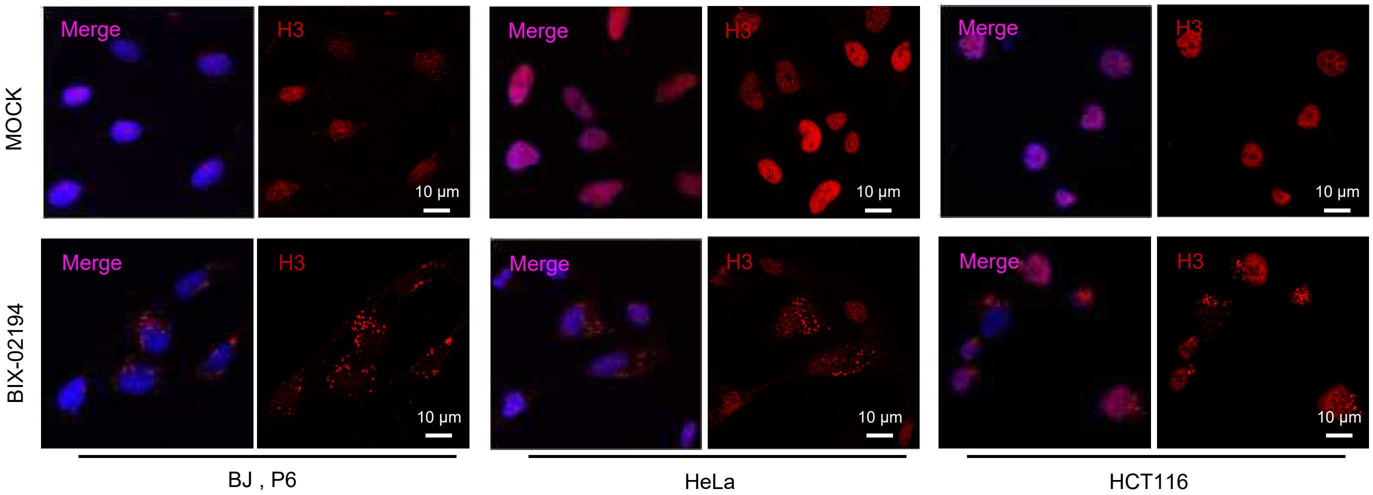

Sup. Fig. 4 BIX-01294 induces cytosolic H3 localization in multiple cell lines. The primary cell line BJ (passage 6), and cancer cell lines HeLa and HCT116 were treated with 5 μM BIX-01294 for 8 hr and analyzed with confocal microscopy. Scale bar, 10 μm.

Sup. Fig. 5

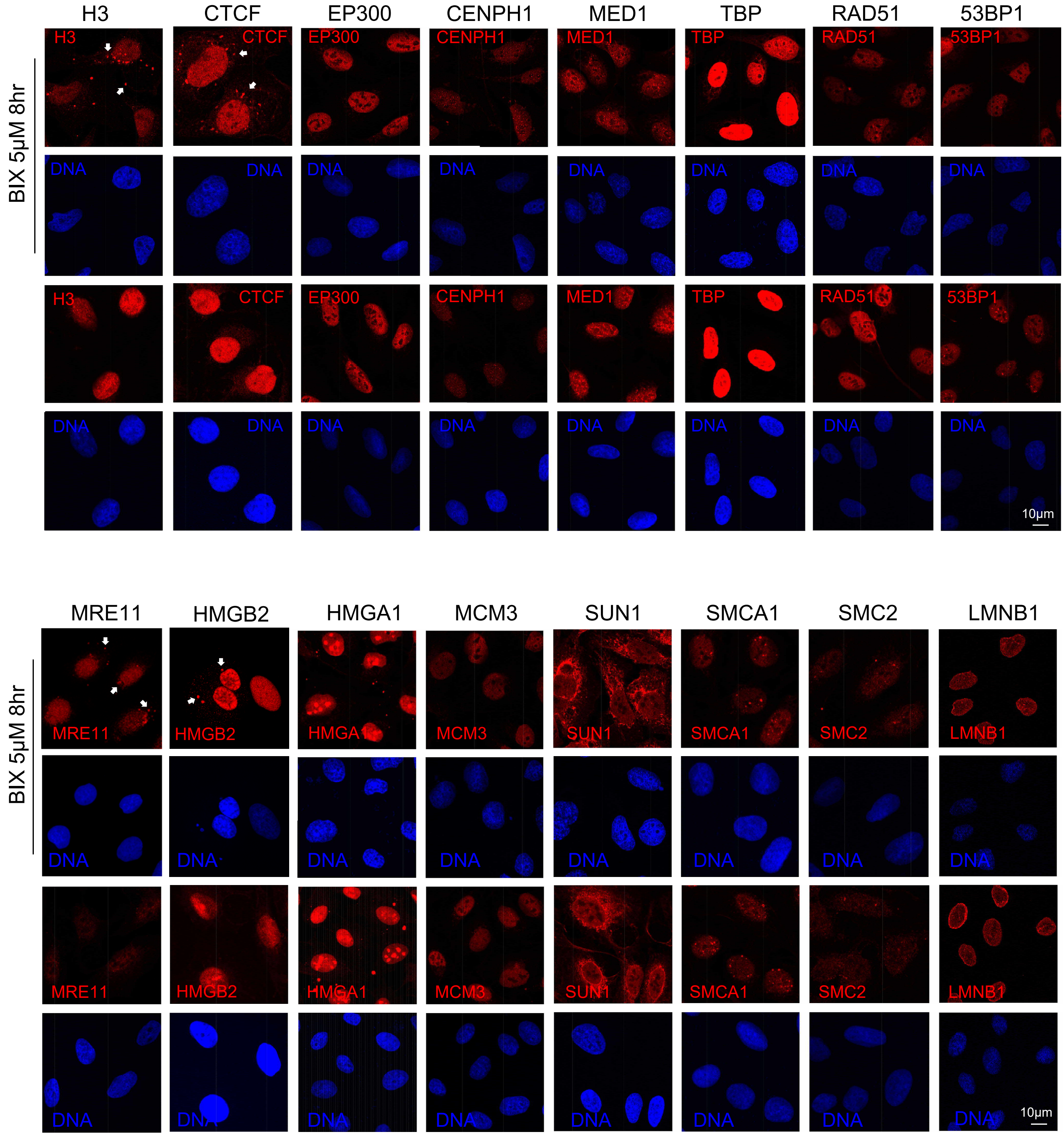

Sup. Fig. 5 Screen for proteins in CLIC puncta. U2OS cells were treated with 5 μM BIX-01294 for 8 hr and stained with indicated antibodies, and imaged with confocal microscopy. Scale bar, 10μm.

Sup. Fig. 6

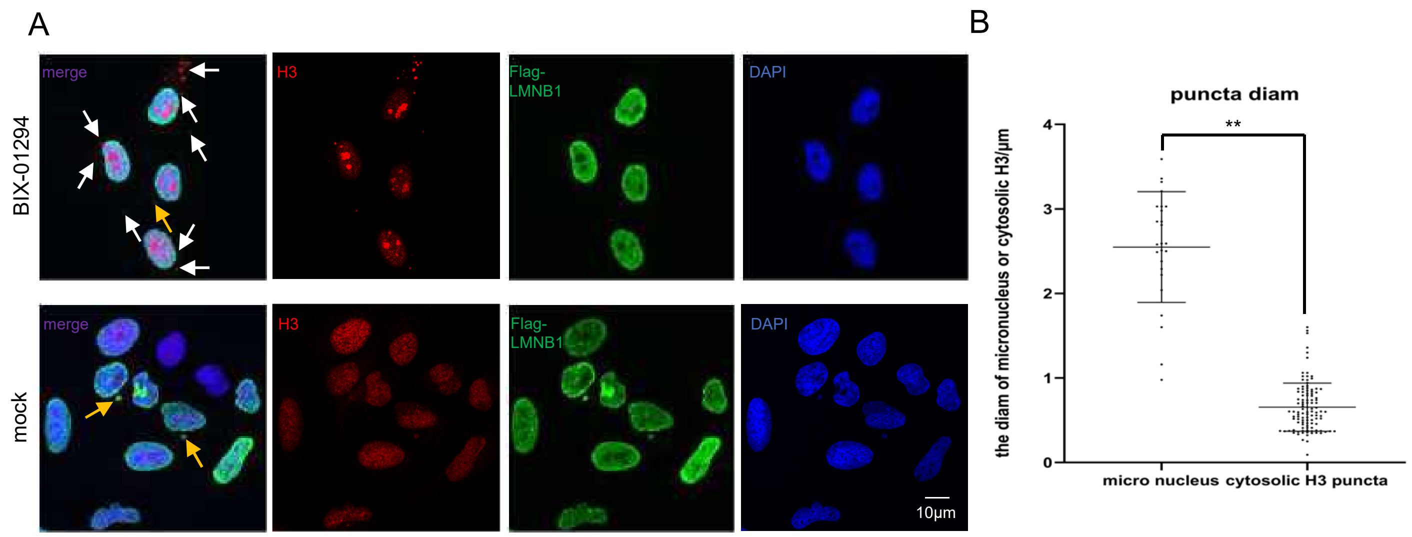

Sup. Fig. 6 The BIX-induced cytosolic histone is different from micronucleus. (A) U2OS cells stably expressing Flag-LMNB1 were treated W/WO BIX-01294, and imaged under a confocal microscope. Scale bar, 10 μm. Cells were treated with 5 μM BIX-01294 for 6 hr. The cytosolic Histone H3 was marked with white arrow, and the micronucleus with yellow arrow. **(B)** Comparison of diameters of micronuclei and CLIC puncta. 24 micronucleus and 108 H3 puncta were selected from 30 random vision fields and their diameters were measured by the LAS AF Lite.** means p value < 0.01.

Sup. Fig. 7

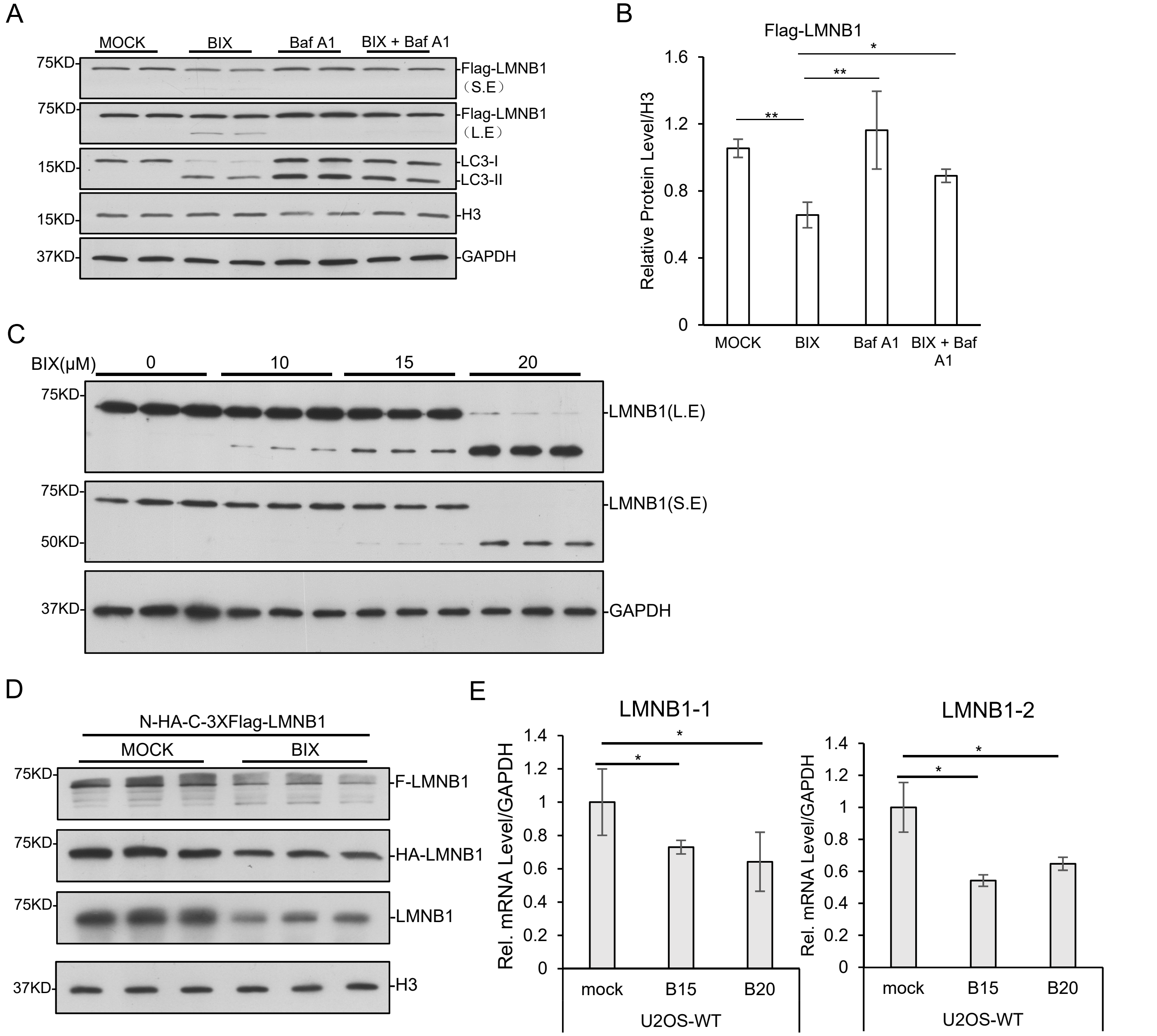

Sup. Fig. 7 BIX-01294 treatment represses LMNB1 at both mRNA and protein levels. (A) Flag-LMNB1 and LC3 in U2OS cell was analyzed by immonoblotting, H3 and GAPDH as loading control. LE, long exposure. SE, short exposure. Cells were treated w/wo 10 μM BIX-01294 for 8 h and 100 nM Baf A1 for 24 hr. **(B)** Densitometric analysis of Flag-LMNB1/GAPDH in (A). **(C)** U2OS cells were treated with BIX-01294 for 8 hr at the indicated concentrations, and analyzed by immunoblotting, GAPDH as loading control. LE, long exposure. SE, short exposure. **(D)** U2OS cells expressing N-HA-C-3XFlag-LMNB1 were treated with 20 μM BIX-01294 for 8 hr and analyzed with immunoblotting, H3 as loading control. **(E)** Quantitative RT-PCR analysis of *LMNB1* in the control and BIX-treated U2OS cells. LMNB1-1 and LMNB-2 represent two different sets of primers. B15, BIX-01294 15 μM for 8 hr; B20, BIX-01294 20 μM for 8 hr. Statistical analyses were performed with t-test. At least three biological replicates were studied for each experiment. * means p value< 0.05; ** means p value< 0.01.

Sup. Fig. 8

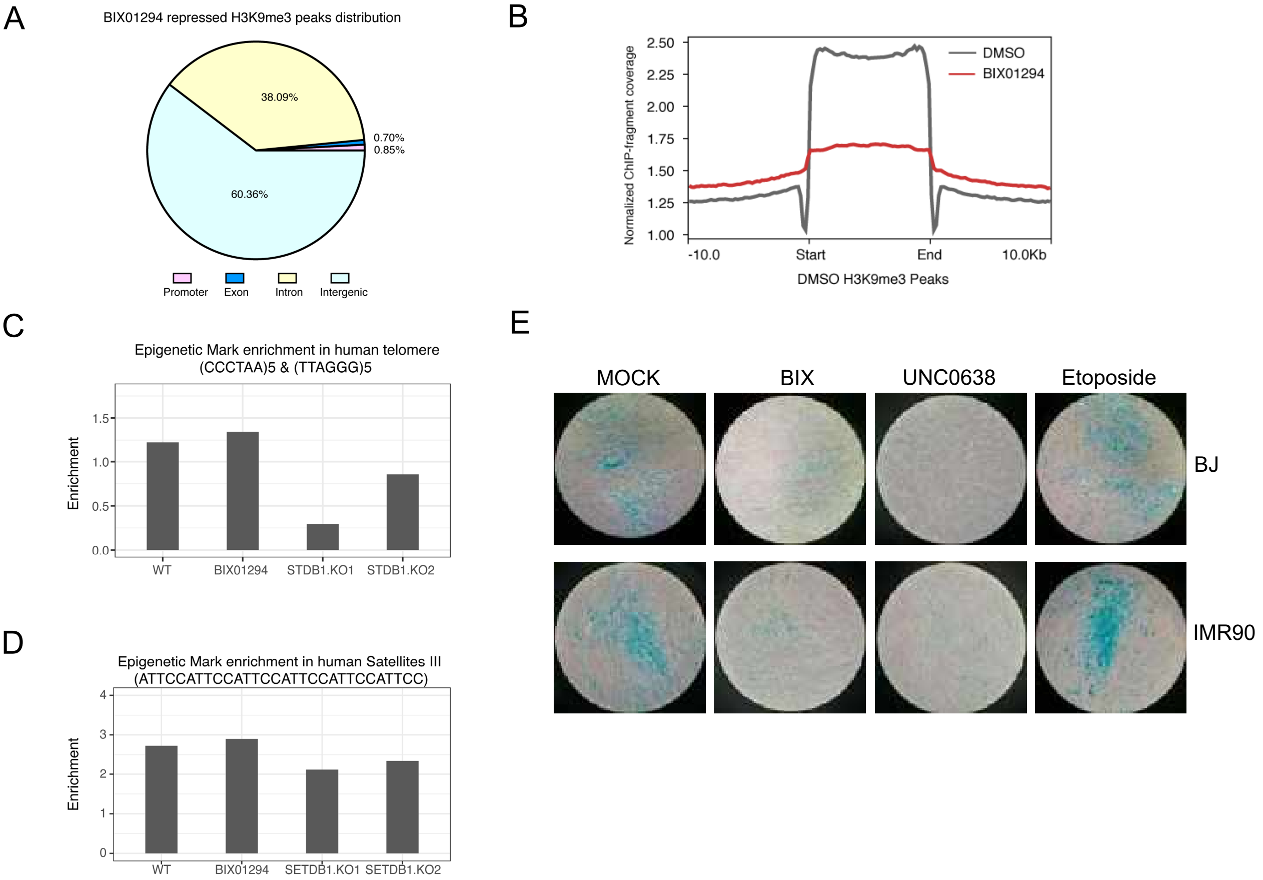

Sup. Fig. 8 BIX-01294 does not induce senescence in cells. (A) The genomic distribution of repressed H3K9me3 peaks by BIX-01294. Cells were treated with 5 μM BIX-01294 for 12 hr. **(B)** Metagene plot representation of the mean H3K9me3 enrichment. The plot was centered at the H3K9me3 peaks in the control cells and the 20kb regions around the peaks are displayed (10kb upstream and 10kb downstream). **(C)** The H3K9me3 enrichment on the telomere sequences [(CCCTAA)5 and (TTAGGG)5] in the mock, BIX-treated, SETDB1 KO-1 and KO-2 cells. Cells were treated with 5 μM BIX-01294 for 12 hr. **(D)** The H3K9me3 enrichment on the satellites III sequemces (ATTCCATTCCATTCCATTCCATTCC in the mock, BIX-treated, SETDB1 KO-1 and KO-2 cells. Cells were treated with 5 μM BIX-01294 for 12 hr. **(E)** Cellular senescence was examined with SA-β-gal staining in primary BJ and IMR90 cells treated with 1 μM BIX-01294, 1 μM UNC0638 or 2 μM Etoposide for 7 Days.

Sup. Fig. 9

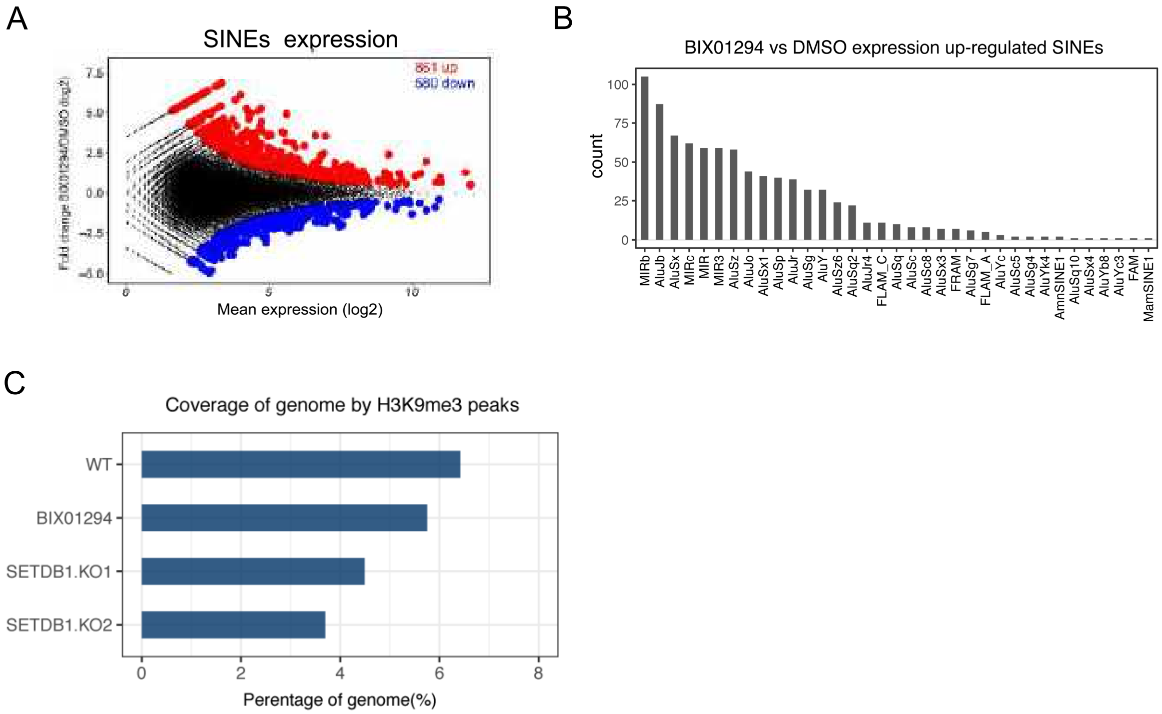

Sup. Fig. 9 BIX-01294 and SETDB1 KO repressed H3K9me3 on SINEs. (A) The scatter plot indicates the RNA levels of SINEs after BIX-01294 treated. **(B)** The top 35 families with the highest number of upregulated elements in (A). **(C)** The genome coverage of H3K9me3 peaks in WT, BIX-01294-treated and SETDB1 KO cells.

Sup. Fig. 10

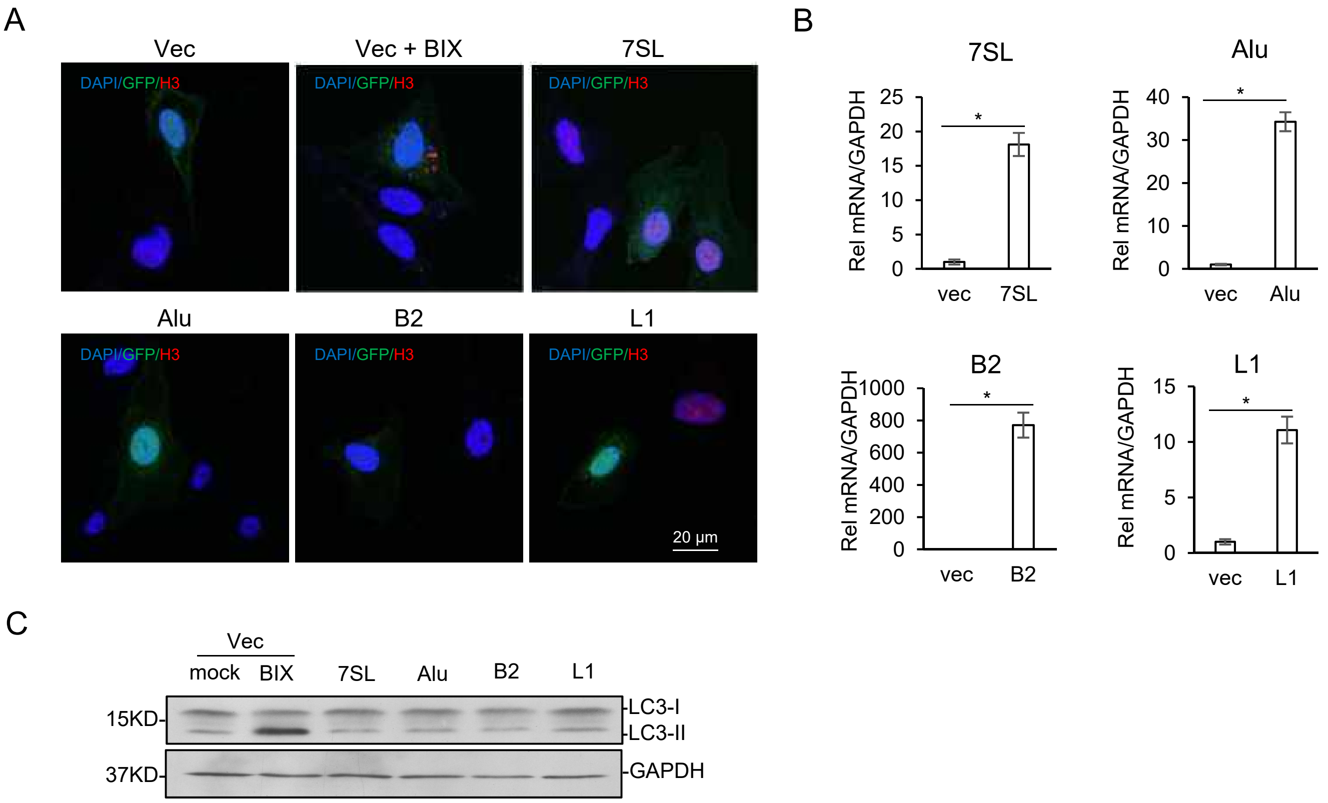

Sup. Fig. 10 Exogenous expression of retrotransposons did not induce CLIC. U2OS cells were transfected with plasmids containing 7SL RNA and RTEs (human SINE Alu, rodent SINE B2 and LINE1), together with GFP, and analyzed with confocal microscopy (**A**, scale bar 20 μm), Q-PCR (**B**, The Q-PCR primer was designed dependent on the specific plasmid promoter) and immunoblotting (**C**, GAPDH as loading control.) BIX, 5 μM for 6hr.

Sup. Fig. 11

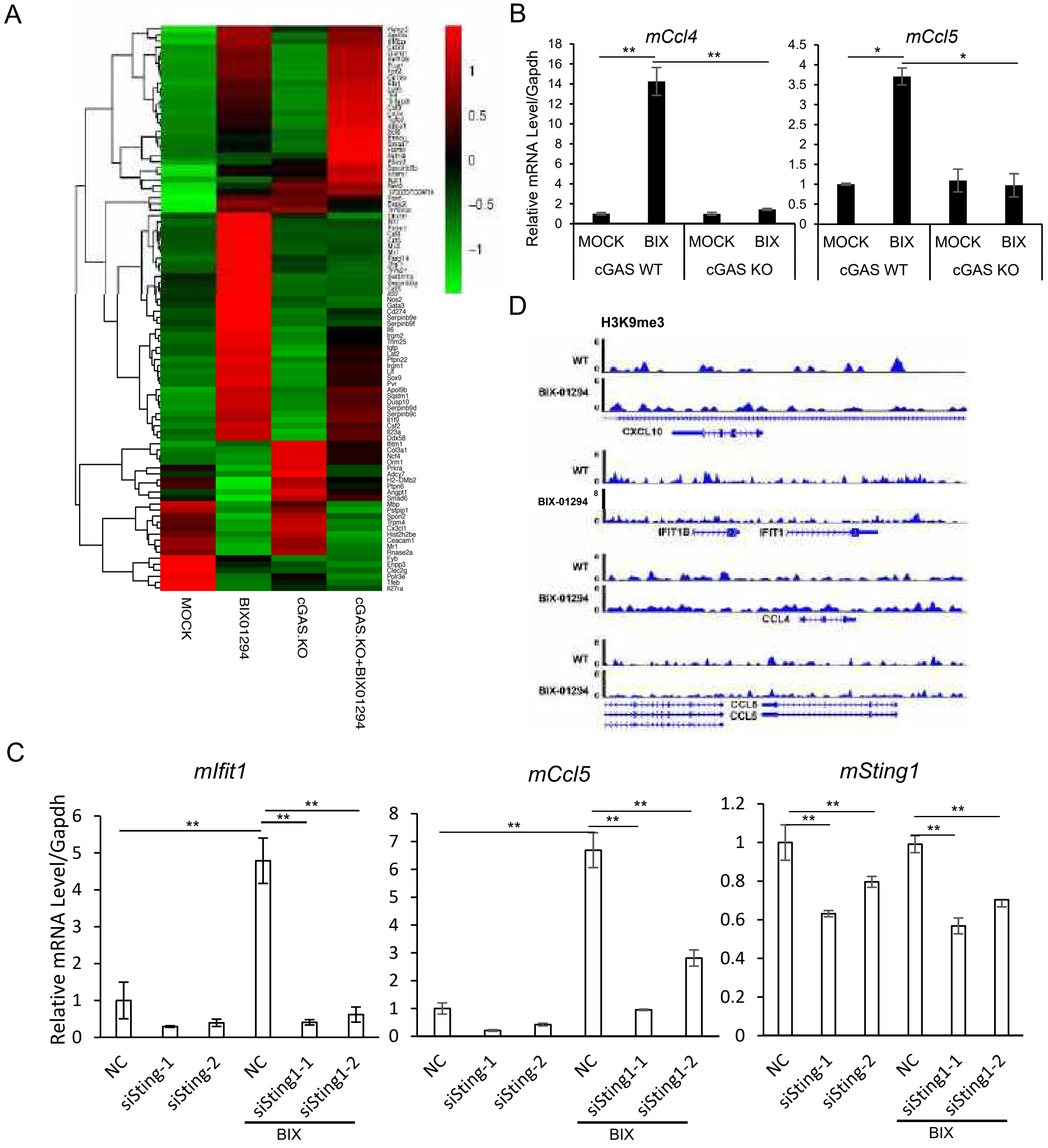

Sup. Fig. 11 BIX-01294 induces cGAS-dependent expression of inflammation and immune related genes. (A) The heat map of BIX-induced inflammatory gene expression in WT and cGAS KO L929 cell lines. **(B)** Quantitative RT-PCR analysis of *Ccl4* and *Ccl5* in the WT and cGAS KO L929 cells. Cells were treated with 10 μM BIX-01294 for 12 hr. **(C)** L929 cells used in Fig. 5B were analyzed with Q-PCR. The mRNA level of *mSting1*, *mIfit1* and *mCcl5* were shown. **(D)** The UCSC genome view of H3K9me3 enrichment on inflammatory genes. H3K9me3 on these genes is all at the background level. Statistical analyses were performed with t-test. At least three biological replicates were studied for each experiment. * means p value< 0.05; ** means p value< 0.01.

Sup. Fig. 12

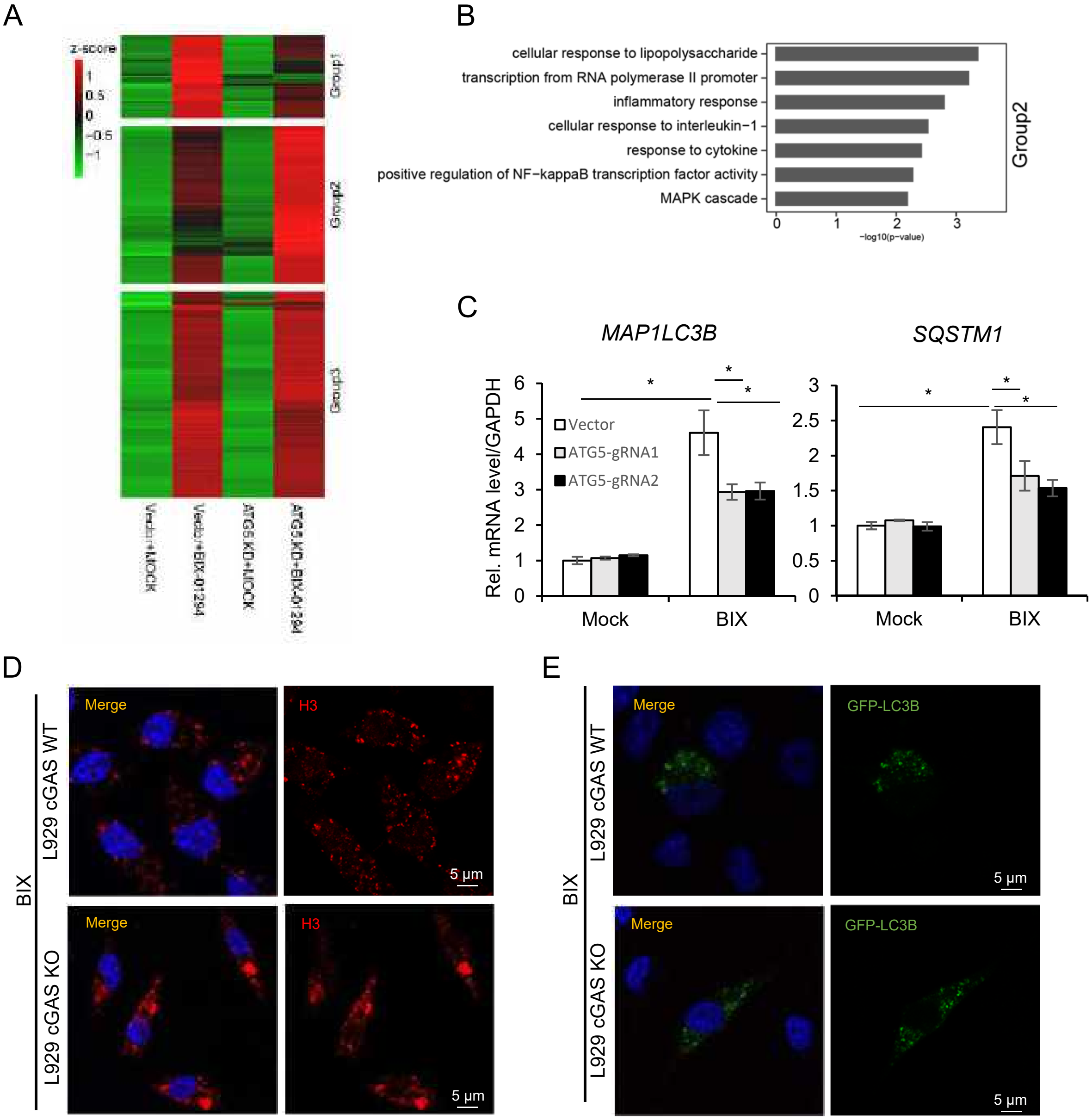

Sup. Fig. 12 A portion of the BIX-01294-induced gene expression is dependent on autophagy. (A) The heat map of upregulated DEGs by BIX-01294 treatment in WT and ATG5 KD U2OS cell line. **(B)** The GO analysis of group 2 in (A) showed that inflammatory genes were further enhanced in ATG5 KD cells. **(C)** Quantitative RT-PCR analysis of MAP1LC3B and SQSTM1/p62 in WT and ATG5 KO cells treated with 5 µM BIX-01294 for 12 hr. Statistical analyses were performed by t-test. **(D&E)** WT and cGAS KO L929 cells were treated with 5 µM BIX-01294 for 8 hr, and H3 (D) and LC3B (E)were imaged with confocal microscopy. Scale bar 5 µm.

Sup. Fig. 13

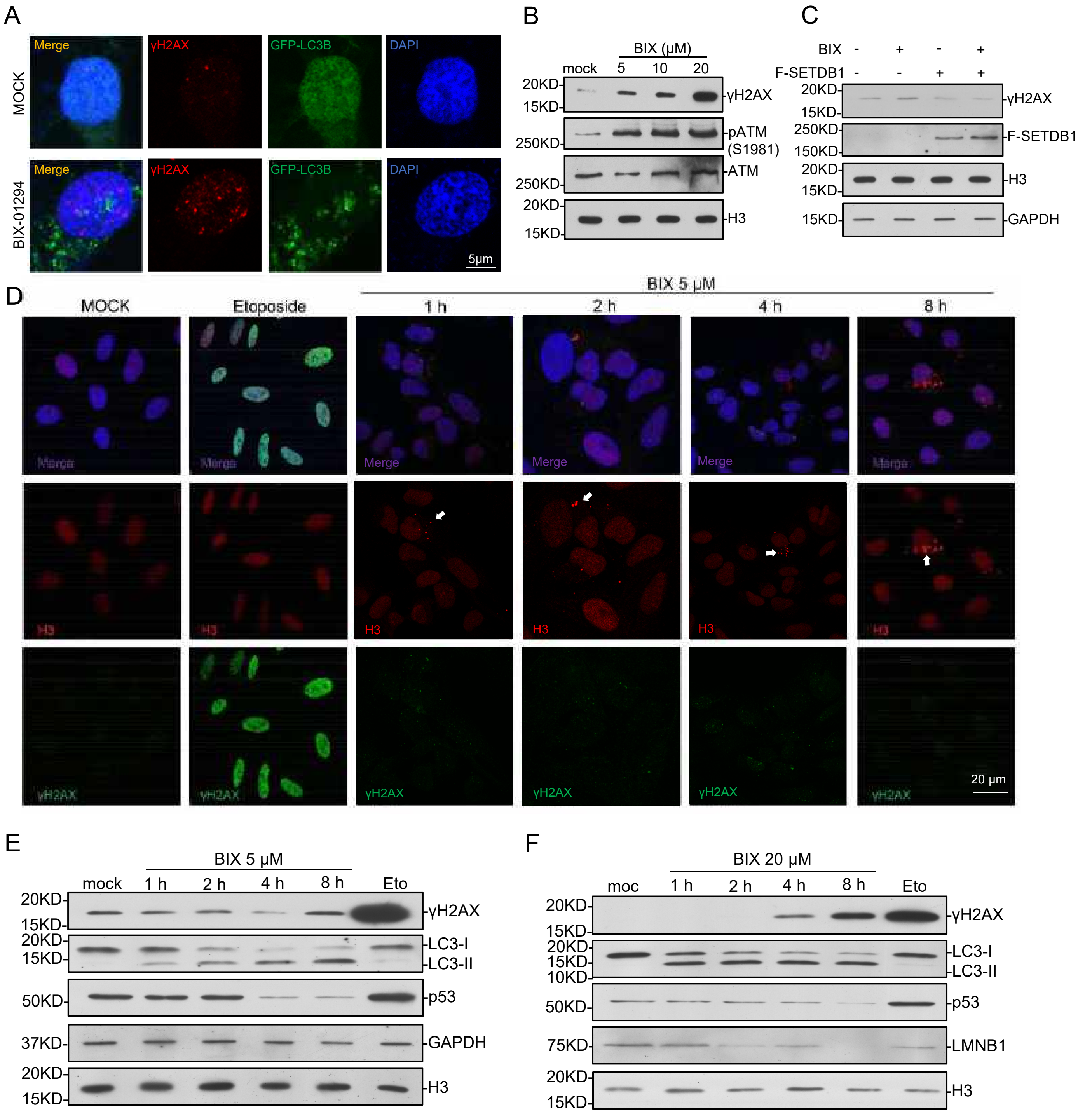

Sup. Fig. 13 H3 translocation to cytoplasm is the upstream event of DNA damage under BIX treatment . (A) GFP-LC3-U2OS cells were treated with 10 µM BIX-01294 for 8 hr, and imaged under a confocal microscope. Scale bar, 5 µm. **(B)** U2OS cells were treated with BIX-01294 for 8 hr at the indicated concentrations, and analyzed by immunoblotting, GAPDH as loading control. **(C)** Control plasmid and Flag-SETDB1 were transfected into U2OS cells treated w/wo 10µM BIX-01294 for 8 hr, and analyzed by immunoblotting, H3 and GAPDH as loading control. **(D).** U2OS cells were treated with 50 µM Etoposide for 8hr or 5 µM BIX-01294 for indicated time, and analyzed with confocal microscopy. White arrows point out CLIC puncta in cells. Scale bar, 20 µm. **(E&F)** U2OS cells were treated with 50 µM Etoposide for 8hr and 5 µM (**E**) or 20µM (**F**) BIX-01294 for indicated time, and analyzed with immunoblotting, H3 as loading contral.

Sup. Fig. 14

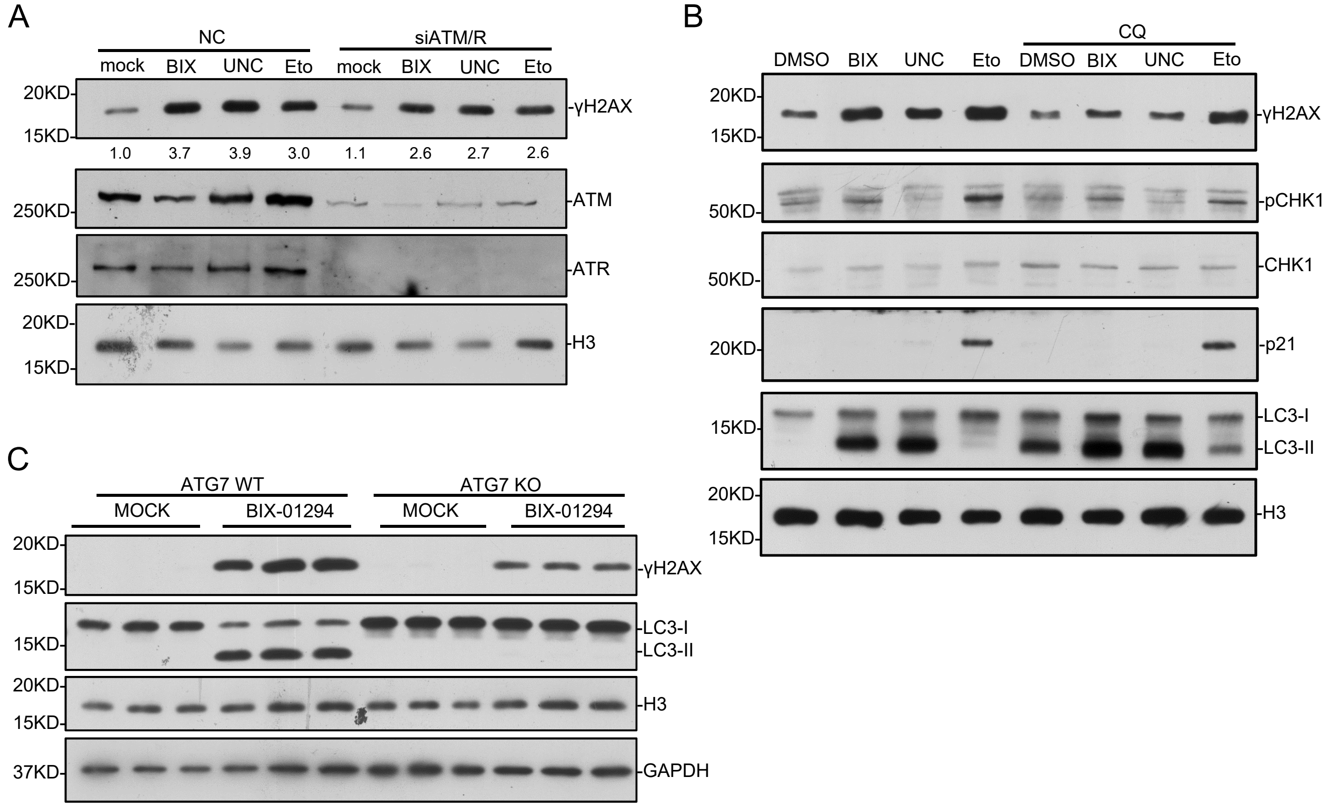

Sup. Fig. S14 Inhibition of autophagy repressed γH2AX induced by H3K9me3 down-regulation. (A) U2OS cells transfected w/wo ATM/ATR siRNA were treated with 20 μM BIX-0294, 20 μM UNC0638 or 50 μM etoposide for 8 hr, and analyzed by immunoblotting, H3 as loading control. **(B)** U2OS cells were treated with 20 μM BIX-01294, 20 μM UNC0638 and 50 μM Etoposide for 8 hr w/wo 20 μM CQ for 24 hr. γH2AX and LC3 in U2OS cells was analyzed with immunoblotting, H3 as loading control. **(C)** WT and ATG7 KO U2OS cells were treated with 20 μM BIX-01294 for 8 hr, and assayed with immunoblotting, H3 and GAPDH as loading control.

Sup. Fig. 15

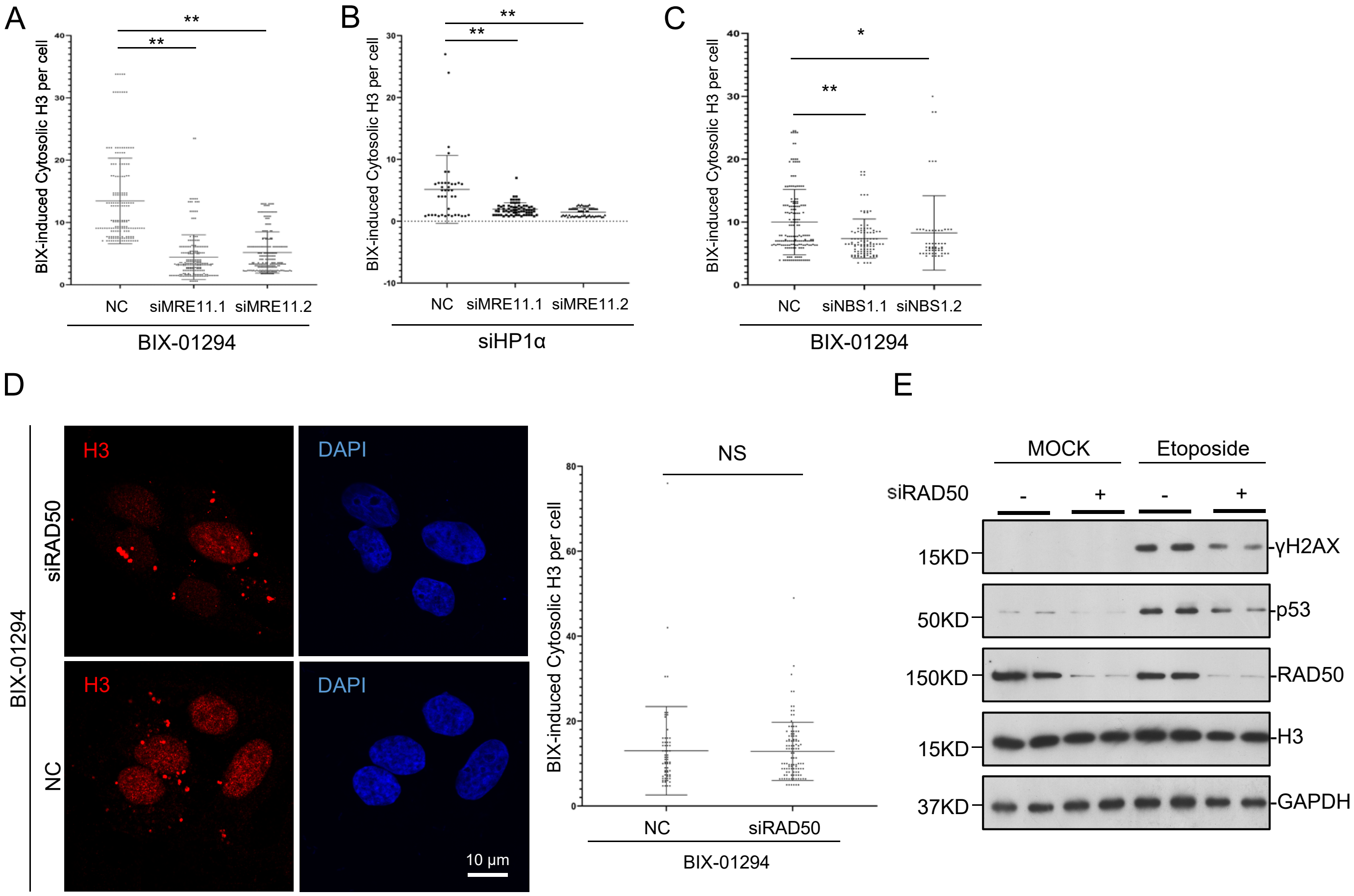

Sup. Fig. 15 RAD50 deficiency does not affect CLIC. (A) The number of CLIC puncta after BIX treatment in Figure 6B was counted manually, and the average numbers were shown (n(NC)=174, n(siMRE11.1) = 221, n(siMRE11.2) = 235). **(B)** The number of CLIC puncta after knocking down HP1α in Figure 6C was counted manually, and the average number was shown (n (NC) = 41, n (siMRE11.1) = 68, n (siMRE11.2) = 57). **(C)** The number of cytosolic H3 puncta after BIX treatment in Figure 6D was counted, and the average number was shown (n (NC)=174, n (siNBS1.1) = 104, n (siNBS1.2) = 55). **(D)** U2OS cells were transfected with control or siRAD50 siRNAs and treated with 5 μM BIX for 8 hr. CLIC puncta were observed with confocal microscopy. Scale bar, 10 μm. **(E)** U2OS cells were transfected with control or siRAD50 siRNAs, treated w/wo 20 μM etoposide for 8 hr and analyzed with immunoblotting, H3 and GAPDH as loading control. Statistical analyses were performed with t-test. * means p value< 0.05. ** means p value< 0.01.

Sup. Fig. 16

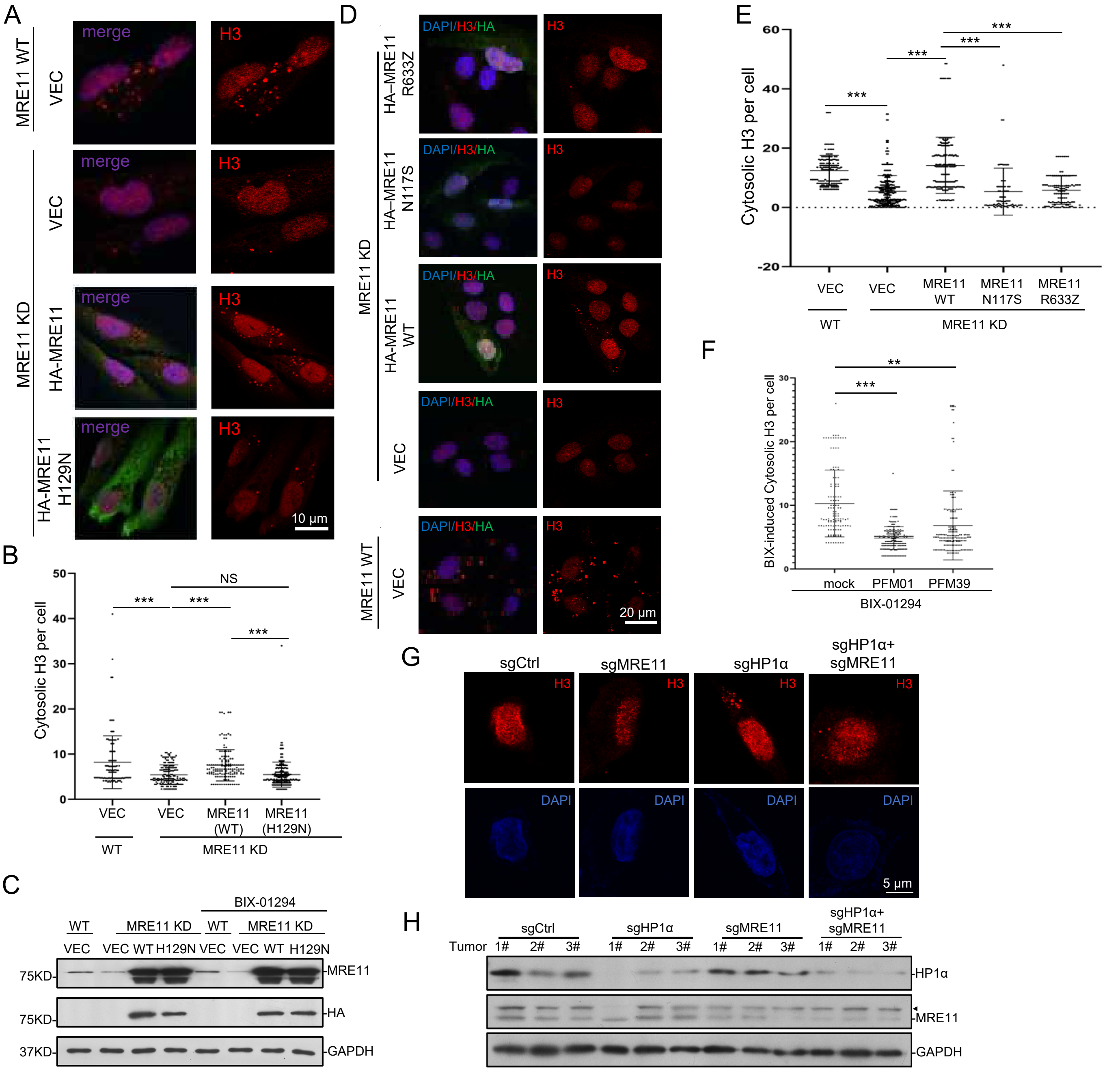

Sup. Fig. 16 MRE11 nuclease activities are required for CLIC. (A) U2OS cells were knocked down with MRE11 sgRNA and then transfected with WT or H129N MRE11 plasmid. Cells were treated with 5µM BIX-01294 for 8 hr, and analyzed with confocal microscopy. Scale bar, 10 µm. **(B)** The number of cytosolic H3 puncta after BIX treatment in (A) was counted and the average number was shown (n (WT) = 116, n (KO) = 170, n (KO+MRE11 WT) = 136, n (KO+MRE11 H129N) = 245). **(C)** Cells used in (A) were assayed with immunoblotting as indicated. **(D)** U2OS cells were knocked down with MRE11 sgRNA and then transfected with WT , N117S or R633Z MRE11 plasmid. Cells were treated with 5µM BIX-01294 for 8 hr, and analyzed with confocal microscopy. Sacale bar, 20 µm. **(E)** The number of CLIC puncta after BIX treatment in (D) was counted, and the average number was shown (n (WT) = 154, n (KD) = 326, n (KD+WT) = 190, n (KD+N117S) = 71, n (KD+H129N) = 159, n (KD+R633Z) = 109). **(F)** The number of cytosolic H3 puncta after BIX treatment in Figure 6E was counted, and the average number was shown (n(mock)=121, n(siPFM01)=218, n(PFM39)=183). **(G&H)** Immunostaining of cells (F) and western blotting of tumors (G) in xenograft experiments. ◂ represents non-specific bands. Statistical analyses were performed with t-test. ** means p value< 0.01,*** means p value< 0.001, NS means Not Significant.
